# Comparative Efficacy of Different AI Systems for Polyp Detection by Size During Colonoscopy: Systematic Review and Network Meta-Analysis

**DOI:** 10.2196/95841

**Published:** 2026-09-09

**Authors:** Ling Ba, Yaxin Qi, Xinrui Lv, Sipu Wang, Lu Yang, Yufeng Wang, Bangmao Wang, Hailong Cao, Xin Xu

**Affiliations:** 1 Department of Gastroenterology and Hepatology Tianjin Medical University General Hospital Tianjin China; 2 Tianjin Yujin Artifcial Intelligence Medical Technology Co.,Ltd. Tianjin China

**Keywords:** artificial intelligence, colonoscopy, colorectal polyp, HKSJ method, network meta-analysis, polyp detection, prediction interval, size-stratified analysis

## Abstract

**Background:**

Colorectal cancer remains a leading cause of death despite being largely preventable through polypectomy. AI systems designed to enhance polyp detection during colonoscopy have shown promise, but the extent to which they improve detection of different-sized polyps remains unclear.

**Objective:**

This study compared the size-stratified efficacy of AI-assisted colonoscopy vs standard colonoscopy using the Hartung-Knapp-Sidik-Jonkman (HKSJ) method, and generated exploratory rankings while acknowledging all cross-platform comparisons are indirect.

**Methods:**

This systematic review and network meta-analysis (NMA) searched PubMed, Embase, Cochrane CENTRAL, and Web of Science from inception to July 25, 2026, supplemented by citation searching. We included randomized controlled trials (RCTs) comparing AI-assisted vs standard colonoscopy in adults (≥18 years of age), reporting mean polyp detection counts stratified by size (≤5 mm, 6-9 mm, and ≥10 mm). Two reviewers screened studies, extracted data, and assessed risk of bias using the Cochrane Risk of Bias 2.0. We conducted frequentist NMA using the HKSJ method with restricted maximum likelihood estimation, calculated 95% prediction intervals (PIs), and assessed heterogeneity using *I*^2^ and τ^2^. Certainty of evidence was rated using the GRADE (Grading of Recommendations Assessment, Development, and Evaluation) framework.

**Results:**

A total of 13 RCTs (4156 participants) compared 8 AI systems to standard colonoscopy, forming a network without direct AI comparisons. For diminutive polyps (≤5 mm), AI showed a modest advantage (standardized mean difference [SMD] 0.21, 95% CI 0.07 to 0.35, 95% PI –1.12 to 1.54), but substantial heterogeneity (*I*^2^=86.6%) and wide PI crossing the null indicated high uncertainty. EndoScreener showed the most consistent evidence (SMD 0.36, 95% CI 0.18-0.54). For small and large polyps, effects were minimal (SMD 0.02, 95% CI –0.02 to 0.06, 95% PI –0.03 to 0.07; SMD 0.01, 95% CI 0.00-0.02, 95% PI –0.01 to 0.03). GRADE certainty was very low for diminutive polyps and low for small and large polyps. Sensitivity analysis excluding Tianjin YuJin did not materially change findings.

**Conclusions:**

AI may modestly enhance diminutive polyp detection, but effects on small and large polyps are minimal, with no platform superiority. Given very low to low certainty, findings are hypothesis-generating. This exploratory NMA provides size-stratified comparisons that can inform future head-to-head trial design. Unlike prior reviews aggregating all polyp sizes, we show the overall AI benefit is driven by diminutive polyp detection, providing a framework for targeted deployment—prioritizing AI for diminutive polyp screening, with limited value for larger lesions. Head-to-head trials are urgently needed.

**Trial Registration:**

PROSPERO International Prospective Register of Systematic Reviews CRD420251266932; https://www.crd.york.ac.uk/PROSPERO/view/CRD420251266932

## Introduction

Colorectal cancer remains the second leading cause of cancer death worldwide, despite being largely preventable through colonoscopic polypectomy [1]. Colonoscopy with polypectomy has been demonstrated to significantly reduce the long-term incidence of colorectal cancer, with studies showing a 65%-67% decrease in a screening population over a median follow-up of 14 years [2]. Furthermore, undergoing endoscopic screening is associated with a substantial reduction in colorectal cancer mortality, and its preventive benefit is independent of and additive to a healthy lifestyle [3]. The protective effect of colonoscopy depends critically on the adenoma detection rate (ADR), an established quality indicator that is inversely associated with the risk of interval colorectal cancer [4,5]. Similarly, as a practical correlate of ADR, higher polyp detection rates (PDR) during colonoscopy are strongly associated with a reduced risk of postcolonoscopy colorectal cancer, with studies demonstrating that a PDR of 42.7% or higher correlates with a 65% relative reduction in cancer incidence compared to lower detection rates [6]. Quality improvement initiatives aimed at enhancing detection, such as audit and feedback programs or advanced imaging technologies, have been shown to significantly increase PDR, thereby potentially lowering the risk of interval colorectal cancers [7]. Yet, even with high-definition white-light endoscopy, a substantial proportion of adenomas, especially small and flat lesions, are missed [8]. Missed lesions arise from 2 main sources: failure to recognize a lesion when it is visible, and incomplete mucosal exposure [9].

AI systems based on deep learning were developed to address the first of these limitations. By superimposing real-time visual alarms on the endoscopic image, AI alerts the endoscopist to suspicious areas, potentially compensating for human perceptual lapses [10,11]. Numerous AI-assisted colonoscopy systems are already in clinical use, and most studies have reported that these systems yield significant increases in ADR, and several meta-analyses have confirmed this benefit [12]. However, the ability of different AI-assisted systems to improve the detection rates of polyps of varying sizes is unknown. There are no head-to-head randomized trials that systematically compare polyp detection performance across all commercially available AI colonoscopy platforms.

Several systematic reviews and meta-analyses have confirmed that AI-assisted colonoscopy enhances adenoma detection and reduces miss rates. Makar et al [13] conducted an updated meta-analysis of 28 randomized controlled trials (RCTs: 23,861 participants), demonstrating a significant 20% increase in ADR and a substantial 55% decrease in adenoma miss rate with computer-aided detection (CADe) systems, with subgroup analyses revealing that the benefit was primarily driven by increased detection of diminutive lesions, while no significant improvement was observed for advanced adenomas [13]. Kumar et al [14] performed a network meta-analysis (NMA) of 17 RCTs (10,547 participants) ranking 5 AI systems (GI Genius, CAD EYE, ENDOANGEL, EndoScreener, and EndoAID) and identified ENDOANGEL as the most effective for ADR [14]. Similarly, a NMA of 64 studies (50,834 participants) by Huang et al [15] demonstrated that ENDOANGEL model-assisted colonoscopy was the most effective method for detecting colorectal adenomas and polyps.

A meta-analysis of 33 RCTs (27,404 patients) by Lou et al [16] confirmed significant increases in both ADR and PDR with AI assistance. Huang et al [15] similarly reported significant improvements in ADR and PDR across 10 RCTs [15]. Most recently, Soleymanjahi et al [17] published a comprehensive meta-analysis confirming that CADe-enhanced colonoscopy increases adenoma detection.

However, several critical knowledge gaps remain. First, none of these NMAs have systematically examined AI efficacy stratified by polyp size (diminutive ≤5 mm, small 6-9 mm, and large ≥10 mm), despite evidence from Makar et al [13] suggesting that overall ADR gains are primarily driven by diminutive lesions and that no significant benefit is observed for advanced adenomas [13]. Second, most existing NMAs used conventional DerSimonian-Laird random-effects models, which may produce inflated type I error rates when the number of studies is small or heterogeneity is substantial. Third, no study has used the Hartung-Knapp-Sidik-Jonkman (HKSJ) method with 95% prediction intervals (PIs) to quantify the clinical impact of heterogeneity and provide more robust estimates for future studies, with the notable exception of Lou et al [16], who used HKSJ adjustment but did not perform size-stratified NMA.

Important gaps also persist regarding interpretive frameworks for NMA outputs. A further key gap, existing NMAs have not explicitly acknowledged that all cross-platform comparisons of AI systems are indirect (via the common comparator of standard colonoscopy), nor have they contextualized size-stratified findings using the GRADE (Grading of Recommendations Assessment, Development, and Evaluation) framework. Finally, no review has systematically examined whether the comparative efficacy of different AI platforms varies by polyp size—a question with direct implications for targeted AI implementation in screening vs surveillance colonoscopy.

Therefore, we conducted this systematic review and NMA to address these gaps. The primary objectives of this study were to compare the efficacy of AI-assisted vs standard colonoscopy for polyp detection, stratified by polyp size (diminutive ≤5 mm, small 6-9 mm, and large ≥10 mm), and to generate exploratory rankings of different AI platforms via surface under the cumulative ranking curve (SUCRA) probabilities, with the explicit acknowledgment that all cross-platform comparisons are indirect. Our prespecified hypotheses were that AI assistance would show size-dependent efficacy, with the greatest benefit observed for diminutive polyps, and that no single AI platform would demonstrate statistically significant superiority over others in indirect comparisons. To improve upon previous reviews [12,18-30], we used the HKSJ random-effects method with 95% PIs to quantify heterogeneity, calculated SUCRA probabilities for exploratory ranking, performed a sensitivity analysis excluding a potentially biased platform (Tianjin YuJin), and assessed the certainty of evidence using the GRADE framework.

## Methods

### Registration

The NMA was conducted according to the PRISMA (Preferred Reporting Items for Systematic Reviews and Meta-Analyses) 2020 statement and the PRISMA extension for NMA (Multimedia Appendices 1-3) [31,32]. The protocol was registered with PROSPERO (International Prospective Register of Systematic Reviews; registration number CRD420251266932). No deviations from the registered protocol occurred.

### Search Strategies

We systematically searched PubMed (via MEDLINE), Cochrane Library (via Wiley), Embase (via Ovid), and Web of Science (Core Collection) from inception to July 25, 2026, for RCTs comparing AI-assisted colonoscopy with standard white-light colonoscopy for polyp detection. The search strategies combined MeSH terms, Emtree terms, free-text words, and Boolean operators, structured around 3 core concepts: population (colonoscopy), intervention (AI), and study design (RCTs), following Cochrane Handbook recommendations. No language or publication date restrictions were applied during the initial database search. The complete search strategies for each database, including all MeSH terms, Emtree terms, field modifiers, Boolean operators, and filters, are provided in Multimedia Appendix 4. Additionally, we performed backward citation searching (screening reference lists of all included studies and relevant reviews) and forward citation searching (using Web of Science and Google Scholar) to identify additional eligible trials. Clinical trial registries (ClinicalTrials.gov and World Health Organization International Clinical Trials Registry Platform) were also searched. The search was updated on July 25, 2026, to capture any new studies published since the initial search. No additional eligible studies were identified.

### Eligibility Criteria

Inclusion criteria were RCTs comparing AI-assisted colonoscopy systems with standard white-light colonoscopy, adult patients (≥18 years of age) undergoing colonoscopy for screening, surveillance, or diagnostic indications, reported outcomes stratified by polyp size (diminutive: ≤5 mm; small: 6-9 mm; and large: ≥10 mm) and English-language full-text articles with sufficient methodological detail for data extraction and risk-of-bias assessment. Studies were grouped for synthesis by polyp size (3 groups).

Exclusion criteria included inflammatory bowel disease or hereditary polyposis syndromes, studies without size-stratified data, nonrandomized designs, and conference abstracts with insufficient methodological details.

### Selection Process

Two reviewers independently screened titles, abstracts, and full texts. Disagreements were resolved by discussion or consultation with a third reviewer. No automation tools were used for screening.

### Data Extraction

Using standardized forms, 2 reviewers independently extracted data from each eligible study: first author, publication year, country, study design, sample size, patient demographics (age and sex), AI system name, and mean number of polyps stratified by size (diminutive: ≤5 mm; small: 6-9 mm; and large: ≥10 mm). For studies reporting multiple AI arms, each AI system was treated as a separate intervention node. Disagreements were resolved by consensus. We did not contact study authors for additional data because all necessary information was available in the published reports. We confirmed that no double counting of participants occurred across studies, as each study contributed independent participants to a single comparison.

### Data Items

For each outcome (mean polyp detection count by size), we gathered all relevant measures, time points, and analyses. When multiple results existed, we prioritized the fully adjusted estimate. We also collected data on participant demographics, intervention details, comparators, and funding sources. Missing or unclear information was considered unreported, and no data were imputed.

### Risk of Bias Assessment

Two reviewers independently assessed the risk of bias in the included studies using the Cochrane Risk of Bias 2.0 (RoB 2.0) tool, which is the current gold-standard tool for assessing bias in randomized trials [33]. The RoB 2.0 tool evaluates bias across 5 domains: bias arising from the randomization process, bias due to deviations from intended interventions, bias due to missing outcome data, bias in measurement of the outcome, and bias in selection of the reported result. For each domain, risk was judged as “low risk,” “some concerns,” or “high risk.” The overall risk of bias for each study was determined based on the domain-level judgments: “low risk” if all domains were low risk, “some concerns” if at least 1 domain had some concerns but no domain was high risk, and “high risk” if any domain was high risk or if multiple domains had some concerns that substantially lowered confidence in the result. Disagreements between reviewers were resolved by discussion or consultation with a third reviewer. Given the nature of endoscopic interventions, blinding of participants and personnel was anticipated to have some concerns or high risk in most studies, which is a common limitation in this field. We acknowledge that our PROSPERO protocol (CRD420251266932) originally specified RoB 1.0; however, we opted to use RoB 2.0 for the present analysis to align with current methodological standards and to strengthen the methodological rigor of our review, as recommended by the editor and recent Cochrane guidance.

### Effect Measures

Primary outcome rationale: we selected mean polyp detection count stratified by size as the primary outcome because it was the only size-stratified measure consistently reported as a continuous variable across included trials. Patient-level detection outcomes such as ADR or PDR were rarely reported in a size-stratified manner, precluding NMA. Standardized mean differences (SMDs) with 95% CIs were therefore calculated. Positive SMD values indicate higher mean polyp detection counts with AI-assisted colonoscopy. We acknowledge that SMD is a relative measure and does not convey absolute incidence; consequently, interpretations regarding surveillance intervals, cost-effectiveness, or platform selection are drawn cautiously and considered exploratory. For ratio measures, we analyzed on the logarithmic scale, but the primary analysis used SMD as all included studies reported continuous outcomes. Across all comparisons, positive SMD values indicate higher mean polyp detection counts in the AI-assisted colonoscopy group relative to standard colonoscopy, reflecting improved detection.

### Synthesis Methods

Because the standard DerSimonian-Laird method can result in inflated type I error rates when the number of studies is small, or heterogeneity is present, we used the HKSJ method for random-effects meta-analyses [34]. In the context of our NMA, the HKSJ method was applied at the pairwise comparison level. For each pairwise comparison between an AI system and standard colonoscopy, the HKSJ method was used to calculate the random-effects pooled estimate and its 95% CI, as implemented in the rma() function of the *metafor* package (version 4.4.3) in R (R Foundation for Statistical Computing) with restricted maximum likelihood (REML) estimation and test=“knha.” This approach accounts for both within-study and between-study variance, providing more conservative CIs when the number of studies is small, or heterogeneity is substantial—conditions that characterize our network, which includes only 13 studies and multiple single-study nodes. Additionally, the HKSJ method was used to calculate 95% PIs for each pooled estimate, quantifying the expected range of true effects in future studies and providing a clinically interpretable measure of heterogeneity impact.

All cross-platform comparisons are indirect (via the common comparator of standard colonoscopy) and were estimated using the *netmeta* package in Stata MP (version 14; Stata Corp); the HKSJ adjustment was applied consistently across both direct and indirect estimates to ensure uniform handling of uncertainty. Critically, this 2-stage HKSJ workflow is statistically valid for our star-shaped evidence network with a single shared standard colonoscopy comparator. All individual AI-vs-control pairwise SMD values were first adjusted via HKSJ within R’s *metafor* package (rma() function, test=“knha,” REML τ^2^ estimation). These fully corrected pairwise effect sizes were then imported into Stata’s netmeta module to generate indirect cross-AI comparative effects. This sequential pipeline retains the conservative CI advantage of HKSJ even for indirect network outcomes, addressing the inflated type I error limitation of traditional DerSimonian-Laird pooling when trial counts are sparse and inter-study heterogeneity is substantial. All 95% PIs reported in this manuscript were exclusively derived from HKSJ-adjusted pooled summary estimates.

The HKSJ method provides more conservative and accurate CIs compared to the standard DerSimonian-Laird approach, particularly when the number of studies is limited, as is the case in our network. Between-study heterogeneity variance (τ^2^) was estimated using REML. To quantify the clinical impact of heterogeneity, we calculated 95% PIs for the summary effect estimates of each comparison (where at least 5 studies were available). The PI provides the range within which the true effect of a future study is expected to lie. We prepared data for synthesis by converting all means, SDs, and sample sizes into SMDs using the *metafor* package in R (version 4.4.3). No missing summary statistics required imputation. We used tabular displays (tables of study characteristics) and graphical methods (forest plots, network plots, SUCRA bar charts, funnel plots, and league tables) to present results. Frequentist NMAs were conducted using the *netmeta* package in Stata MP 14.

The network geometry was visualized with node size proportional to sample size and edge thickness to the number of studies. Consistency was evaluated using a global consistency test via a design-by-treatment interaction model and local consistency via a loop-specific approach. Inconsistency was defined as *P*<.10. Because the network was star-shaped with no direct head-to-head trials between AI systems, the consistency assumption could not be formally tested for loops; we therefore relied on the global test across designs (all *P*>.05). SUCRA was estimated to provide exploratory treatment hierarchies. Because the network is star-shaped with no direct head-to-head trials, SUCRA values are presented as exploratory only and are not interpreted as evidence of superiority. To explore possible causes of heterogeneity, we performed prespecified subgroup analyses by polyp size (the primary grouping). Meta-regression was not performed due to the limited number of studies. We also conducted sensitivity analyses excluding the Tianjin YuJin system (due to potential conflict of interest). Using alternative effect measures (risk difference) to test robustness (results not materially different, data not shown).

### Reporting Bias Assessment

We assessed funnel plot asymmetry to examine small-study effects, which may arise from publication bias, selective outcome reporting, true heterogeneity, or chance [35]. Funnel plots and Egger linear regression test were used when ≥10 studies were available. Asymmetry was considered significant at *P*<.10. Importantly, funnel plot asymmetry should not be equated with publication bias alone, as small-study effects can result from other factors such as differences in study methodology, true variation in effect estimates across study sizes, or random variation. We therefore interpreted the results of the Egger test cautiously, recognizing that significant asymmetry indicates the presence of small-study effects rather than definitively confirming publication bias.

### Certainty Assessment

We assessed the overall certainty of evidence for the primary outcomes (mean polyp detection count stratified by size) using the GRADE framework (Multimedia Appendix 5). The certainty was rated as high, moderate, low, or very low based on 5 domains: risk of bias, inconsistency (including width of the 95% PI), indirectness, imprecision, and publication bias. GRADE assessments were performed independently by 2 reviewers, with disagreements resolved by consensus. Data and code availability: all extracted data are provided in Multimedia Appendix 6. The R code for HKSJ meta-analysis, PIs, and figures is available in Multimedia Appendix 7.

### Ethical Considerations

This systematic review and NMA used published data from previously approved RCTs; no primary data collection or patient contact was required. The protocol was registered with PROSPERO (CRD420251266932) and conducted according to PRISMA 2020 guidelines.

## Results

### Study Selection and Characteristics

**Figure 1:**
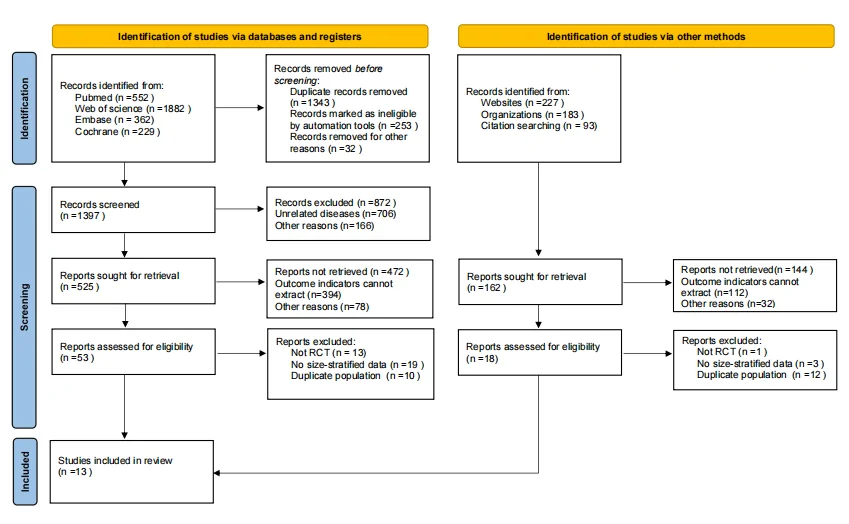
PRISMA (Preferred Reporting Items for Systematic Reviews and Meta-Analyses) flow diagram showing the systematic literature search and study selection process for the network meta-analysis of AI-assisted colonoscopy systems. RCT: randomized controlled trial.

The literature search identified 3247 records, of which 13 RCTs comprising 4156 participants were included in the NMA (Figure 1). After removing duplicates, 2 reviewers screened titles and abstracts, followed by full-text assessments. A total of 13 RCTs with 4156 participants were included in the NMA. Only English-language full-text articles met the inclusion criteria, as non-English records lacked sufficient methodological detail or were conference abstracts with incomplete data. A total of 7 studies were conducted in China, 2 in the United States, and 1 each in Japan, the United Kingdom, and international multicenter settings. Overall, 8 AI-assisted systems were evaluated: EndoScreener (n=3), EndoAngel (n=2), CAD EYE (n=2), AI-assisted pooled systems (n=2), and single studies for Wision AI, Tianjin YuJin, GI Genius, and Olympus. Standard white-light colonoscopy served as the common comparator in all studies; no head-to-head trials directly compared AI systems (Table 1). An updated search performed on July 25, 2026, identified 1247 new records. After full-text review, no additional studies met the inclusion criteria.

**Table 1:** Characteristics of included randomized controlled trials (RCTs) comparing AI-assisted colonoscopy with standard colonoscopy.

Study ID	AI system	First author	Year	Research type	Centers	Author states	Sample size (AI-assisted/standard colonoscopy)
1	Olympus	Thomas Ka-Luen Lui [18]	2024	RCT	Multicenter	Hong Kong, China	238/214
2	EndoScreener	Pu Wang [19]	2020	RCT	Monocentric	China	484/478
3	CAD EYE	Daisuke Yamaguchi [20]	2023	RCT	Multicenter	Japan	113/118
4	CAD EYE	Hirotaka Nakashima [21]	2023	RCT	Monocentric	Japan	207/208
5	EndoScreener	Jeremy R Glissen Brown [22]	2021	RCT	Multicenter	United States	113/110
6	GI Genius	Ahmir Ahmad [23]	2023	RCT	Monocentric	United Kingdom	308/306
7	Wision AI	Pu Wang [24]	2019	RCT	Monocentric	China	522/536
8	Tianjin YuJin	Ping Shen [25]	2021	RCT	Multicenter	China	64/64
9	EndoAngel	Liwen Yao [26]	2022	RCT	Monocentric	China	268/271
10	EndoScreener	Peixi Liu [27]	2020	RCT	Monocentric	China	393/397
11	AI-assisted	Lei Xu [28]	2021	RCT	Multicenter	China	1177/1175
12	AI-assisted	Liu WN [29]	2019	RCT	Monocentric	China	508/518
13	EndoAngel	Dexin Gong [30]	2020	RCT	Monocentric	China	355/349

### Network Geometry and Consistency

**Figure 2:**
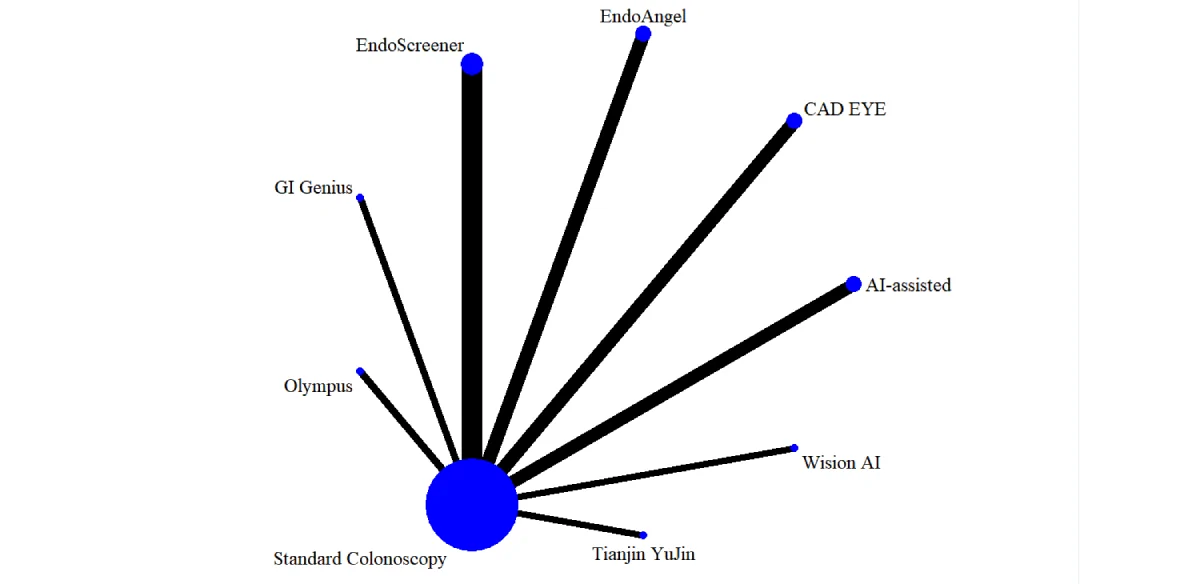
Network geometry plot illustrating the connections between AI-assisted colonoscopy systems and standard colonoscopy. Node size is proportional to sample size, and edge thickness represents the number of direct comparative studies. No direct head-to-head comparisons exist between AI systems. Therefore, all comparisons between AI systems are indirect, and rankings should be interpreted as exploratory. No head-to-head trials between Al systems. All comparisons are indirect via standard colonoscopy.

The network formed a star-shaped structure with standard colonoscopy as the central common comparator. A total of 8 AI-assisted systems connected directly to standard colonoscopy, with no direct connections between AI systems (Figure 2). Global consistency tests revealed no significant inconsistency across size-stratified networks (all *P*>.05), but local consistency could not be evaluated due to the lack of closed loops, relying instead on clinical plausibility.

### Size-Stratified Mean Polyp Detection Count Analysis

A random-effects NMA using the HKSJ method with REML estimation was performed. Exploratory SUCRA values were calculated for hypothesis generation, but these rankings should not be seen as evidence of clinical superiority due to the sparse network and indirect comparisons. It is important to emphasize that these SUCRA rankings are derived solely from indirect comparisons and should not be interpreted as evidence of superiority of one AI system over another. The rankings are provided for hypothesis-generating purposes only, to help prioritize which AI systems should be compared in future head-to-head trials. No clinical recommendations can be made based on these rankings.

### Diminutive Polyps (≤5 mm)

**Figure 3:**
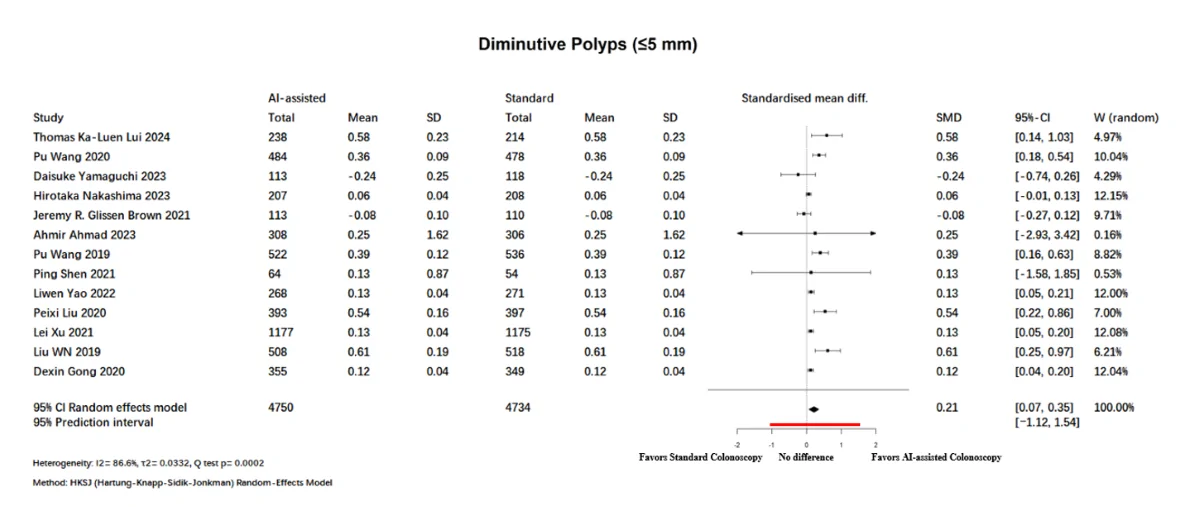
Forest plot of standardized mean difference (SMD) for mean count of diminutive polyps (≤5 mm), comparing AI-assisted colonoscopy vs standard colonoscopy. Positive SMD values indicate higher polyp-detection counts in the AI-assisted group (right side of the x-axis, favors AI-assisted colonoscopy); negative SMD values indicate higher detection counts in the standard-colonoscopy group (left side of the x-axis, favors standard colonoscopy). Pooled estimates were obtained using the Hartung-Knapp-Sidik-Jonkman (HKSJ) random-effects model. Black horizontal bars represent 95% CIs for individual studies; the thick red horizontal line represents the 95% prediction interval for future studies. The black diamond denotes the pooled SMD effect size. Heterogeneity statistics (I2, τ2, and Q-test *P* value) are reported at the bottom of the plot [18-30].

**Figure 4:**
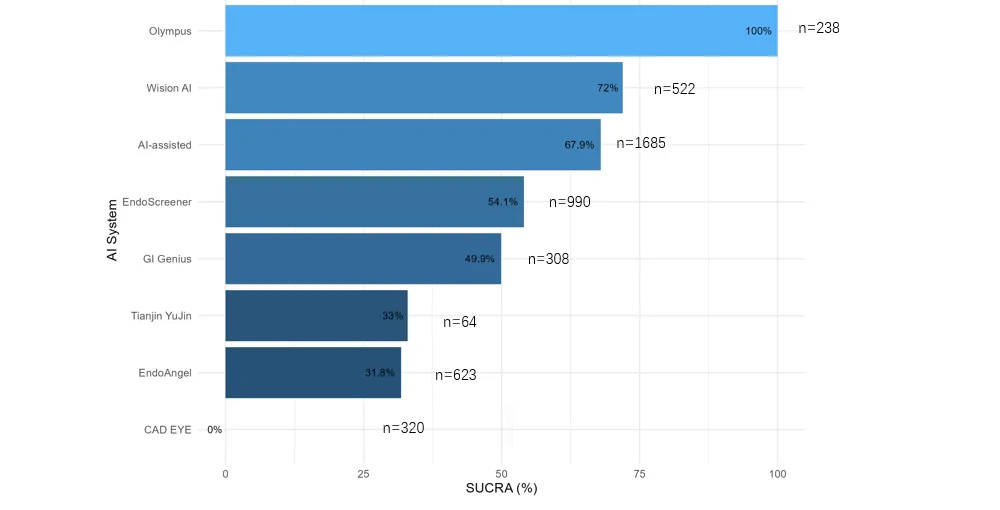
Exploratory surface under the cumulative ranking curve (SUCRA) probabilities for diminutive polyps (≤5 mm). SUCRA values are based solely on indirect comparisons via standard colonoscopy; no head-to-head trials exist between AI systems. Rankings are exploratory only and should not be interpreted as evidence of superiority. Based only on indirect comparisons; no head-to-head trials. Rankings are exploratory only.

This NMA enables indirect comparative efficacy evaluation across all 8 AI colonoscopy systems, an analytical output that cannot be obtained from separate pairwise meta-analyses alone. NMA of 11 study arms comparing 7 AI systems vs standard colonoscopy demonstrated significant heterogeneity in AI system efficacy for increasing diminutive polyp counts (*I*^2^=86.6%; τ^2^=0.0332). The pooled SMD indicated a slight advantage for AI-assisted colonoscopy (SMD 0.21, 95% CI 0.07-0.35), but the wide 95% PI (–1.12 to 1.54) crosses the null threshold and includes negative SMD values, demonstrating that AI-assisted colonoscopy may deliver no polyp detection benefit, or even inferior performance compared to standard white-light colonoscopy in specific patient cohorts, endoscopic workflows, or operator groups. EndoScreener was associated with a statistically significant increase in mean polyp detection count compared with standard colonoscopy. Indirect comparisons between AI systems revealed no statistically significant differences (Figure 3 [18-30]). SUCRA values are presented for hypothesis generation only and should not be interpreted as evidence of clinical superiority due to the absence of direct head-to-head comparisons between AI systems. Rankings are based solely on indirect comparisons via standard colonoscopy. Olympus ranked highest (100%), followed by Wision AI (72%), AI-assisted pooled systems (67.9%), EndoScreener (54.1%), GI Genius (49.9%), Tianjin YuJin (33%), EndoAngel (31.8%), and CAD EYE (0%; Figure 4). Values represent SMDs (95% CIs) for row intervention minus column intervention. All comparisons are indirect via standard colonoscopy; no head-to-head trials exist between AI systems. Nonsignificant comparisons are indicated by CIs crossing zero (Figure S4 in Multimedia Appendix 8). According to GRADE, the certainty of this evidence was very low, downgraded for risk of bias (unblinded operators in 12/13, 92.3% of studies), serious inconsistency (*I*^2^=86.6%, 95% PI crossing the null), and indirectness (predominantly Asian populations). All pooled estimates were obtained using the HKSJ random-effects method with REML estimation.

### Small Polyps (6-9 mm)

**Figure 5:**
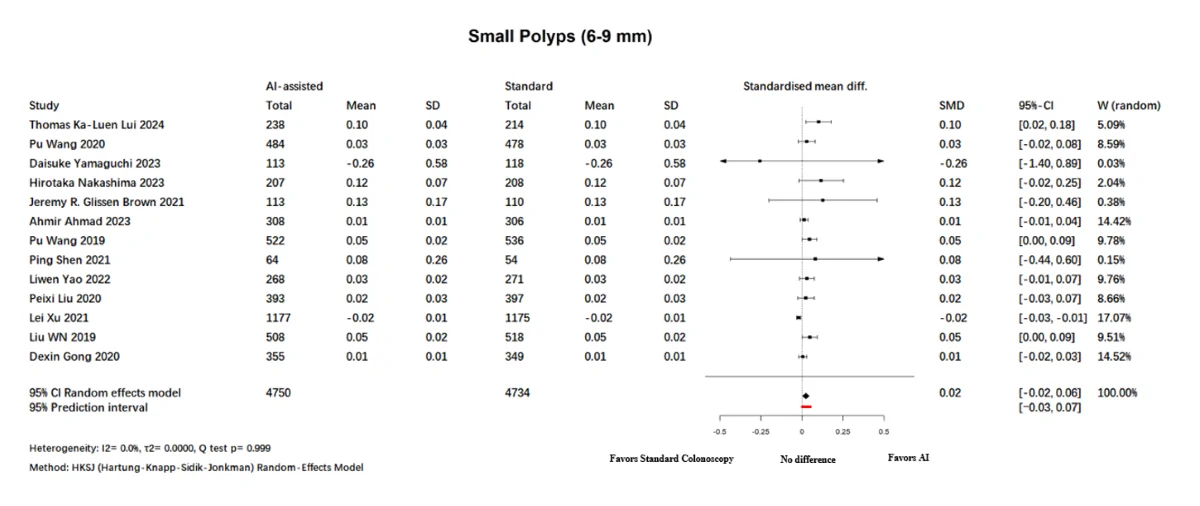
Forest plot of standardized mean difference (SMD) for mean count of small polyps (6-9 mm), comparing AI-assisted colonoscopy vs standard colonoscopy. Positive SMD values indicate higher polyp-detection counts in the AI-assisted group (right side of the x-axis, favors AI-assisted colonoscopy); negative SMD values indicate higher detection counts in the standard-colonoscopy group (left side of the x-axis, favors standard colonoscopy). Pooled estimates were obtained using the Hartung-Knapp-Sidik-Jonkman (HKSJ) random-effects model. Black horizontal bars represent 95% CIs for individual studies; the thick red horizontal line represents the 95% prediction interval for future studies. The black diamond denotes the pooled SMD effect size. Heterogeneity statistics (I2, τ2, and Q-test *P* value) are reported at the bottom of the plot [18-30].

**Figure 6:**
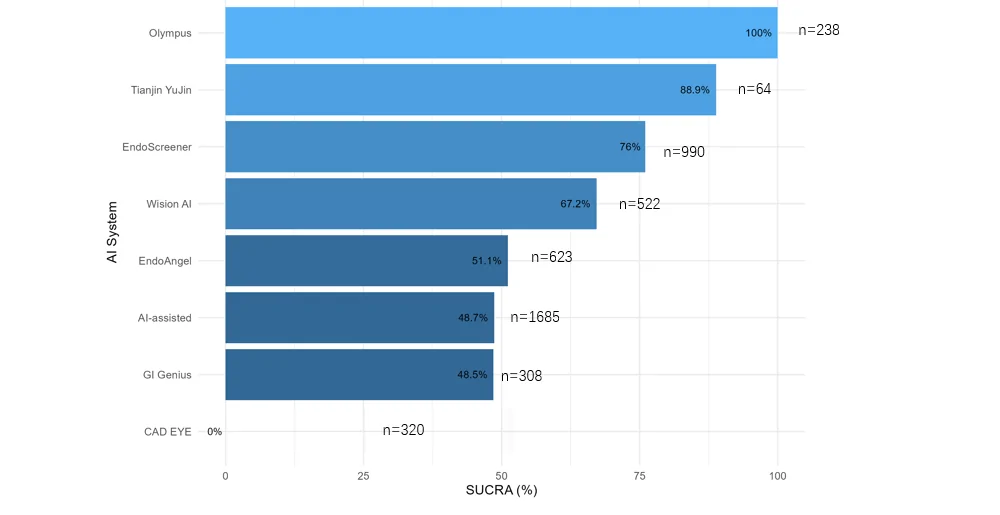
Exploratory surface under the cumulative ranking curve (SUCRA) probabilities for small polyps (6-9 mm). SUCRA values are based solely on indirect comparisons via standard colonoscopy; no head-to-head trials exist between AI systems. Rankings are exploratory only and should not be interpreted as evidence of superiority. Based only on indirect comparisons; no head-to-head trials. Rankings are exploratory only.

This NMA enables indirect comparative efficacy evaluation across all 8 AI colonoscopy systems, an analytical output that cannot be obtained from separate pairwise meta-analyses alone. For small polyps (6-9 mm), effect estimates were near null, and SUCRA rankings varied, with Olympus again leading. The lack of direct trials and minimal pooled effect prevent definitive conclusions on platform performance for small polyps. We advise against viewing high exploratory SUCRA values for any system as evidence of clinical superiority for small polyps, as this may be due to higher baseline detection rates, varying size definitions, operator learning, or statistical uncertainty (Figure 5 [18-30]). Olympus ranked highest (100%), followed by Tianjin YuJin (88.9%), EndoScreener (76%), Wision AI (67.2%), EndoAngel (51.1%), AI-assisted pooled systems (48.7%), GI Genius (48.5%), and CAD EYE (0%). These rankings should not be interpreted as evidence of clinical superiority (Figure 6). All comparisons are indirect via standard colonoscopy. The minimal effect sizes and overlapping CIs across all comparisons indicate no significant differences between AI systems for small polyp detection (Figure S6 in Multimedia Appendix 8). The certainty of this evidence was low according to GRADE, downgraded for risk of bias and indirectness. The 95% PI was –0.03 to 0.07, confirming that the near-null average effect is consistent across settings. The narrow 95% PI (–0.03 to 0.07) also spans the null value of zero, meaning consistent polyp detection superiority of AI cannot be guaranteed across diverse real-world clinical settings; null or inferior detection outcomes are plausible in some trial populations.

### Large Polyps (≥10 mm)

**Figure 7:**
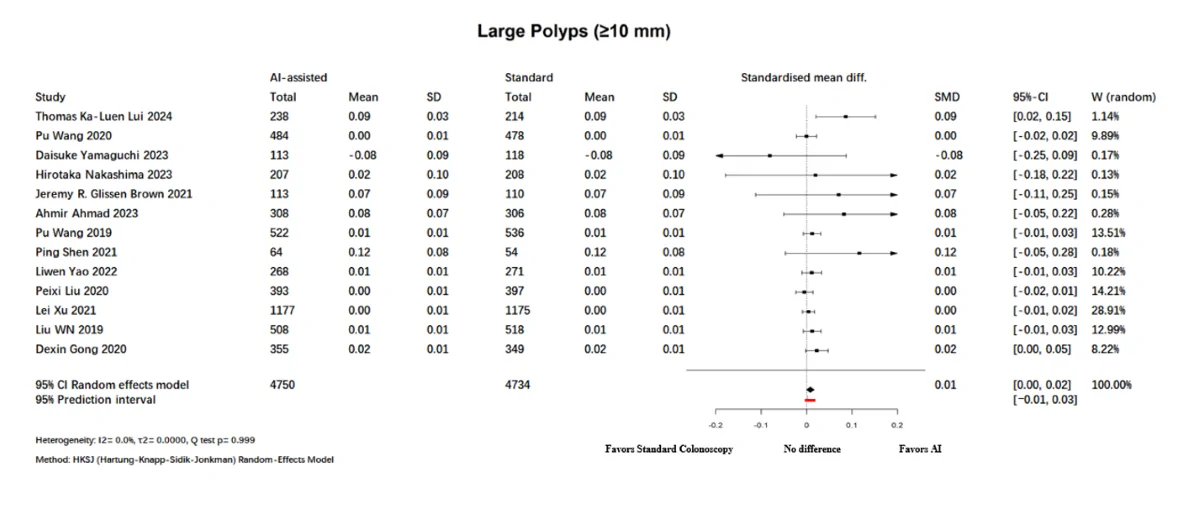
Forest plot of standardized mean difference (SMD) for mean count of large polyps (≥10 mm), comparing AI-assisted colonoscopy vs standard colonoscopy. Positive SMD values indicate higher polyp-detection counts in the AI-assisted group (right side of the x-axis, favors AI-assisted colonoscopy); negative SMD values indicate higher detection counts in the standard-colonoscopy group (left side of the x-axis, favors standard colonoscopy). Pooled estimates were obtained using the Hartung-Knapp-Sidik-Jonkman (HKSJ) random-effects model. Black horizontal bars represent 95% CIs for individual studies; the thick red horizontal line represents the 95% prediction interval for future studies. The black diamond denotes the pooled SMD effect size. Heterogeneity statistics (I2, τ2, and Q-test *P* value) are reported at the bottom of the plot [18-30].

**Figure 8:**
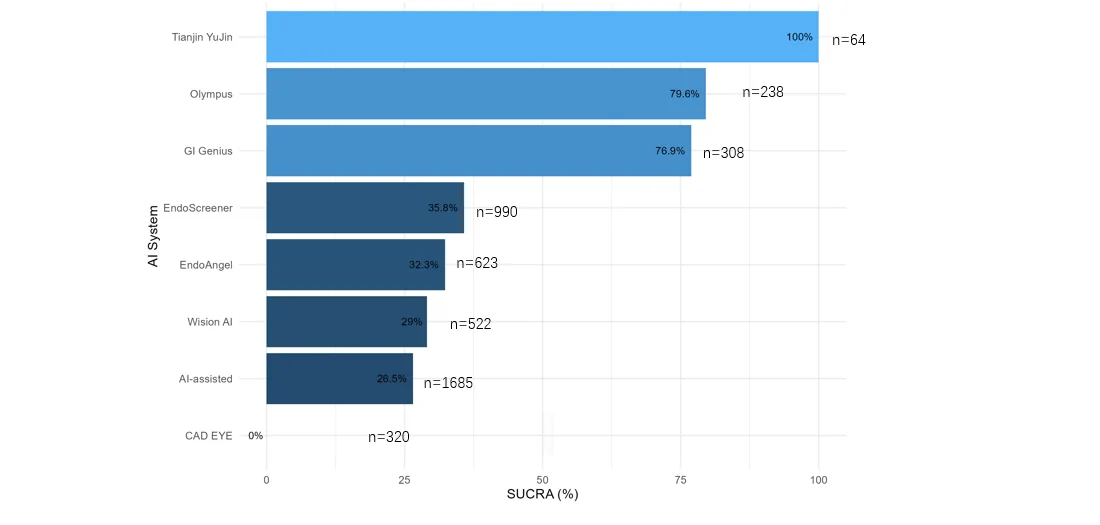
Exploratory surface under the cumulative ranking curve (SUCRA) probabilities for large polyps (≥10 mm). SUCRA values are based solely on indirect comparisons via standard colonoscopy; no head-to-head trials exist between AI systems. Rankings are exploratory only and should not be interpreted as evidence of superiority. Based only on indirect comparisons; no head-to-head trials. Rankings are exploratory only.

This NMA enables indirect comparative efficacy evaluation across all 8 AI colonoscopy systems, an analytical output that cannot be obtained from separate pairwise meta-analyses alone. For large polyps (≥10 mm), no AI system showed significant improvement over standard colonoscopy (pooled SMD 0.01, 95% CI 0.00-0.02). Tianjin YuJin had the highest SUCRA (100%), followed by Olympus (79.6%) and GI Genius (76.9%), but this ranking is based on a single small study (n=128) and should not be seen as proof of clinical superiority. A sensitivity analysis excluding Tianjin YuJin, due to potential bias, did not change the overall finding, confirming no meaningful AI advantage for large polyps. The minimal SUCRA separation and overlapping CIs suggest that AI assistance adds limited value for large, more visible lesions (Figure 7 [18-30]). Tianjin YuJin ranked highest (100%), followed by Olympus (79.6%), GI Genius (76.9%), EndoScreener (35.8%), EndoAngel (32.3%), Wision AI (29%), AI-assisted pooled systems (26.5%), and CAD EYE (0%). Note that the top ranking of Tianjin YuJin is derived from a single small study (n=128) and does not constitute evidence of clinical superiority (Figure 8). The near-zero effect estimates and wide CIs preclude definitive conclusions regarding relative efficacy of different AI systems for large polyp detection (Figure S5 in Multimedia Appendix 8)**.** The certainty of this evidence was low according to GRADE, downgraded for imprecision (few studies contributing data) and indirectness. The 95% PI was –0.01 to 0.03, indicating that the absence of benefit is consistent across settings. The 95% PI (–0.01 to 0.03) crosses zero, consistent with the near-null pooled SMD; AI assistance provides no reliable incremental polyp detection advantage for large lesions across heterogeneous clinical environments.

**Figure 9:**
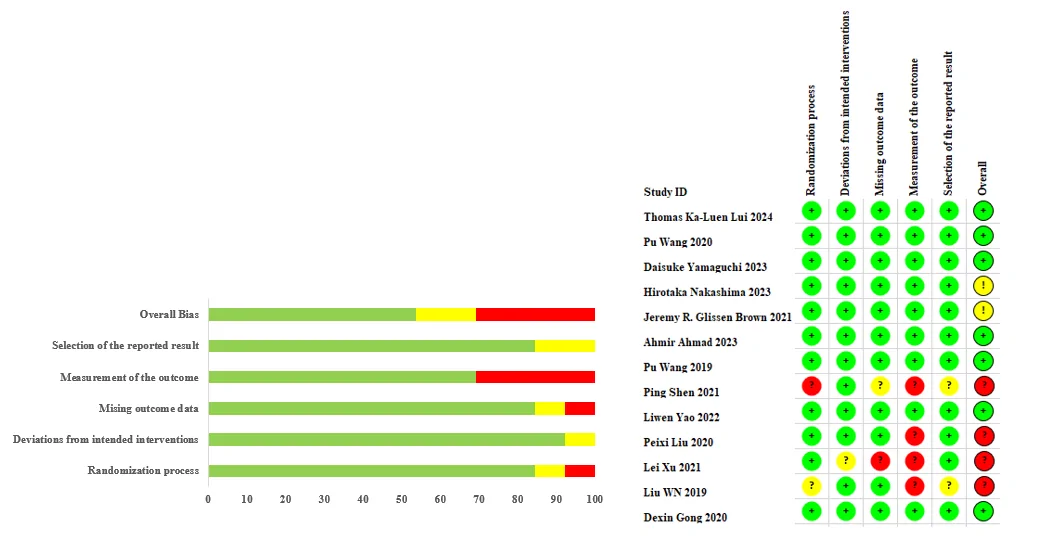
Summary plot of risk of bias assessment across all included studies using the Cochrane Risk of Bias Tool 2.0. The color coding corresponds to the following risk of bias judgments: green=low risk, yellow=some concerns, and red=high risk. The 7 domains assessed were random sequence generation, allocation concealment, blinding of participants and personnel, blinding of outcome assessment, incomplete outcome data, selective reporting, and other bias. Overall risk of bias for each study was judged as low if all domains were low risk, some concerns if any domain was unclear or high risk, and high risk if any domain was rated high risk [18-30].

### Sensitivity Analysis

Excluding the Tianjin YuJin system did not significantly alter findings across polyp sizes. The pooled SMD for diminutive polyps remained stable, and estimates for small and large polyps were unchanged (Figures S1-S3 in Multimedia Appendix 8). The sensitivity analysis excluding Tianjin YuJin confirmed that the exclusion did not materially change any pooled estimates, suggesting that the potential conflict of interest did not introduce systematic bias. However, the GRADE certainty remained unchanged (very low for diminutive, and low for small and large polyps), as the primary limitations—unblinded operators and heterogeneity-were inherent to the included studies rather than driven by a single trial.

### Risk of Bias Assessment

**Figure 10:**
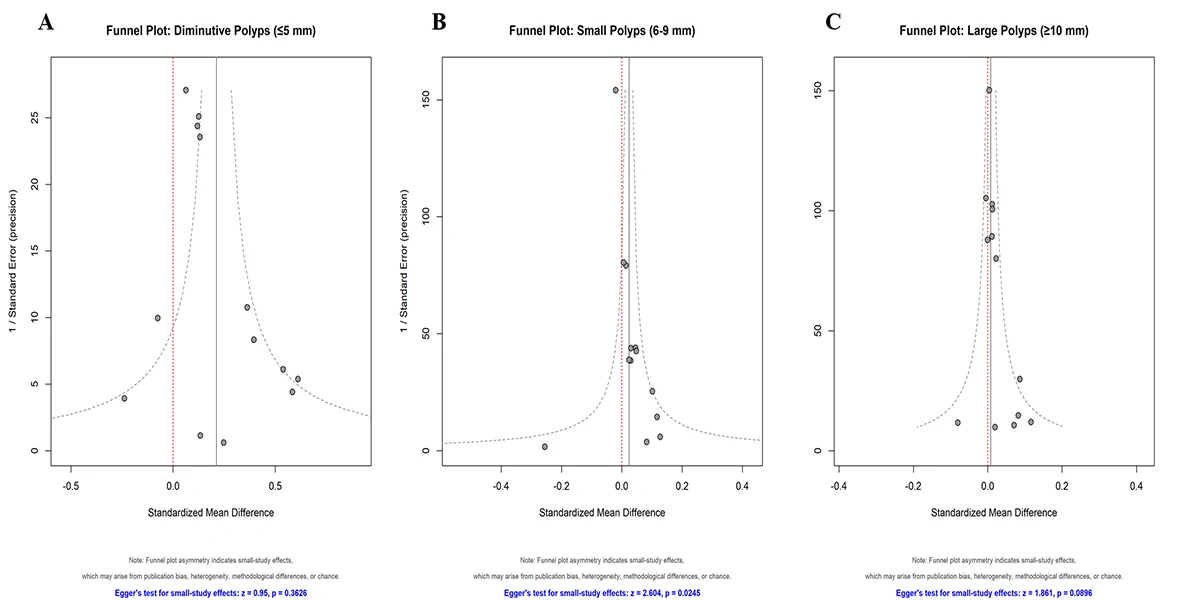
(A) Funnel plot for assessing small-study effects (as an indicator of potential reporting bias) for diminutive polyps (≤5 mm). Egger test *P*=.36. (B) Funnel plot for assessing small-study effects for small polyps (6-9 mm). Egger test *P*=.03. (C) Funnel plot for assessing small-study effects for large polyps (≥10 mm). Egger test *P*=.09. Funnel plot asymmetry may arise from multiple factors, including true heterogeneity between studies, chance, methodological differences across studies of varying sample sizes, or reporting biases such as publication bias or selective outcome reporting. Consistent with Cochrane guidance, funnel plot asymmetry is interpreted as evidence of small-study effects and is not equated with publication bias alone.

No new significant indirect comparisons between AI platforms were found. Risk of bias was assessed using the Cochrane RoB 2.0 tool across 5 domains for each included study (Figure 9 [18-30] and Table 2). Across the 13 included trials, the overall risk of bias was judged as low in 2 (15.4%) studies, as having some concerns in 5 (38.5%) studies, and as high in 6 (46.2%) studies. The most common source of bias was the “deviations from intended interventions” domain, which was rated as high risk or having some concerns in the majority of studies due to the inherent inability to blind endoscopists and participants to the AI intervention. This is a methodological constraint intrinsic to AI-assisted colonoscopy trials rather than a marker of poor study conduct. The “measurement of the outcome” domain was predominantly low risk, as polyp detection counts were objectively measured and histopathologically confirmed. The “selection of the reported result” domain showed some concerns in 5 (38.5%) studies, primarily due to insufficient preregistration of analysis plans. Funnel plots and Egger test were used to assess small-study effects, which could result from factors like publication bias or true heterogeneity (Figure 10 [18-30]). The limitations identified in the RoB 2.0 assessment were fully considered in the GRADE certainty assessment and in the interpretation of all findings.

**Table 2:** Risk of bias summary for included studies (Cochrane Risk of Bias [RoB] 2.0)^a^.

Study	Randomization process	Deviations from intended interventions	Missing outcome data	Measurement of the outcome	Selection of the reported result	Overall bias
Lui et al, 2024 [18]	Low	Low	Low	Low	Low	Low
Wang et al, 2020 [19]	Low	Low	Low	Low	Low	Low
Yamaguchi et al, 2023 [20]	Low	Low	Low	Low	Low	Low
Nakashima et al, 2023 [21]	Low	Low	Low	Low	Low	Some concerns
Glissen Brown et al, 2021 [22]	Low	Low	Low	Low	Low	Some concerns
Ahmad et al, 2023 [23]	Low	Low	Low	Low	Low	Low
Wang et al, 2019 [24]	Low	Low	Low	Low	Low	Low
Shen et al, 2021 [25]	High	Low	Some concerns	High	Some concerns	High
Yao et al, 2022 [26]	Low	Low	Low	Low	Low	Low
Liu et al, 2020 [27]	Low	Low	Low	High	Low	High
Xu et al, 2021 [28]	Low	Some concerns	High	High	Low	High
Liu et al, 2019 [29]	Some concerns	Low	Low	High	Some concerns	High
Gong et al, 2020 [30]	Low	Low	Low	Low	Low	Low

^a^The “Deviations from intended interventions” domain was rated as high risk in most studies due to the inherent inability to blind endoscopists and participants to the AI intervention, which is an intrinsic limitation of AI-assisted colonoscopy trials rather than poor study conduct. The “Measurement of the outcome” domain was predominantly low risk as polyp detection counts were objectively measured and histopathologically confirmed. Overall risk of bias was determined per RoB 2.0 guidance: “Low” if all domains were low risk; “Some concerns” if at least 1 domain had some concerns but no domain was high risk; “High” if any domain was high risk or if multiple domains had some concerns that substantially lowered confidence in the result.

### GRADE Certainty Assessment

We assessed the certainty of evidence for the primary outcomes using the GRADE framework (Table 3). For diminutive polyps (≤5 mm), the certainty was rated as very low, downgraded for risk of bias (unblinded operators in most studies), substantial inconsistency (*I*^2^=86.6%, wide 95% PIs), and indirectness (predominantly Asian populations). For small polyps (6-9 mm), certainty was low, downgraded for risk of bias and indirectness. For large polyps (≥10 mm), certainty was low, primarily due to imprecision from sparse data and indirectness. No outcome achieved moderate or high certainty, reflecting the methodological limitations of the current evidence base.

**Table 3:** GRADE (Grading of Recommendations Assessment, Development, and Evaluation) assessment of outcome measures.

Certainty assessment								Patients, n			Effect size		Certainty		Importance
Studies, n	Study design	Risk of bias	Inconsistency	Indirectness	Imprecision	Other considerations	Intervention		Control	SMD^a^ (95% CI)					
**Mean number of diminutive polyps detected (≤5 mm)**															
13	Randomized trials	Serious^b^	Very serious^c^	Not serious	Not serious	None	4750		4734	0.21 (0.07 to 0.35)		Very low^b,c^		Critical	
**Mean number of small polyps detected (6-9 mm)**															
13	Randomized trials	Serious^b^	Not serious	Not serious	Serious^d^	None	4750		4734	0.02 (–0.02 to 0.06)		Low^b,d^		Critical	
**Mean number of large polyps detected (** **≥** **10 mm)**															
13	Randomized trials	Serious^b^	Not serious	Not serious	Serious^e^	None	4750		4734	0.01 (0.00 to 0.02)		Low^b,e^		Critical	

^a^SMD: standardized mean difference.

^b^Most trials lacked endoscopist blinding; partial inadequate allocation concealment.

^c^*I*^2^=86.6%, 95% prediction interval crosses null, large between‑study heterogeneity.

^d^Effect estimate close to null; wide CI crossing no‑effect.

^e^Effect estimate close to the null; trivial clinical magnitude.

### Small-Study Effects Assessment

Egger test showed significant small-study effects for small polyps (*P*=.03), borderline for large polyps (*P*=.09), and none for diminutive polyps (*P*=.36). With only up to 13 studies and a fragmented network, these tests have low statistical power, and the observed asymmetry should be interpreted cautiously. As discussed in the Methods section, funnel plot asymmetry is indicative of small-study effects, which may reflect publication bias, selective outcome reporting, true heterogeneity, chance, or differences in methodological quality across studies. Therefore, while we cannot exclude the possibility of reporting bias, the observed asymmetry may also be attributable to the limited number of small studies, true variation in effect estimates, or chance. We did not interpret these results as definitive evidence of publication bias (Figure 10).

## Discussion

This NMA reveals marked size-dependent efficacy of AI-assisted colonoscopy systems for increasing mean polyp detection counts, with distinct patterns for diminutive, small, and large lesions. The analysis indicates a consistent pattern: AI assistance may offer a modest incremental benefit for diminutive polyps, yet this potential benefit weakens for small polyps and is negligible for large polyps overall. Any observable advantage is limited to smaller lesions, whereas little measurable improvement can be expected for larger polyps.

Our size-stratified findings extend prior meta-analyses by demonstrating that the overall benefit of AI in colonoscopy varies considerably by lesion size, a heterogeneity that conventional aggregate analyses have largely obscured. Recent tandem RCTs (same-day sequential dual colonoscopies performed on identical participants to quantify within-patient polyp miss rates via direct intraindividual comparison) have confirmed that computer-aided colonoscopy significantly reduces adenoma miss rates compared with white-light colonoscopy alone [36]; yet, such analyses rarely examine whether this protective effect differs across polyp sizes. Our study represents the first NMA to apply robust heterogeneity-aware analytical workflows for AI-assisted colonoscopy with stratification by polyp dimension. However, a major limitation of this approach is the absence of direct head-to-head trials, meaning that all cross-platform comparisons are indirect. We explicitly acknowledge this limitation throughout the manuscript and frame our findings as exploratory and hypothesis-generating rather than definitive.

Although the absence of direct head-to-head trials limits the strength of the NMA findings, the NMA still provides valuable information that standard pairwise meta-analyses cannot offer. First, it synthesizes all available evidence into a single coherent framework, enabling indirect comparisons between AI systems that have never been directly compared in clinical trials. Second, it generates exploratory rankings that can inform the design of future head-to-head randomized trials by identifying which AI systems warrant further investigation. Third, it provides a comprehensive overview of the evidence landscape, revealing gaps in the literature and highlighting areas where direct evidence is most urgently needed. These contributions are explicitly acknowledged as exploratory and hypothesis-generating, not as evidence of comparative superiority.

The modest effect observed for diminutive polyps must be interpreted cautiously. First, considerable between-study variation indicates that observed benefits may not translate uniformly across clinical settings, and some populations may derive no practical improvement. Second, the high risk of bias due to unblinded operators in most studies may have inflated the effect estimate through performance bias, as endoscopists aware of AI activation might exert extra effort during procedures. Third, available study evidence imposes constraints on interpretive confidence, and future research is highly likely to change these findings. For small and large polyps, study observations point toward minimal practical added value, but the quality of available evidence still warrants caution in interpretation.

Similarly, while NMAs have established the superiority of CADe over chromoendoscopy and ranked individual AI systems by overall performance [13], these comparisons rely on an assumption of uniform treatment effects across lesion categories, an assumption our findings challenge. Kumar et al [14] identified ENDOANGEL as the most effective system for adenoma detection. However, these rankings were derived from overall ADR without stratification by polyp size. Similarly, an NMA of 64 studies by Huang et al [15] ranked ENDOANGEL as the most effective for adenoma and polyp detection, yet did not examine whether system performance varied across lesion sizes. The importance of such stratified examination resonates with methodological advances in precision medicine; for instance, recent NMAs in oncology have similarly revealed that treatment hierarchies for immunotherapy vary substantially across patient subgroups defined by programmed death-ligand 1 expression and histology, underscoring that aggregate comparisons may mask clinically meaningful heterogeneity [14].

Recent methodological commentaries emphasize that AI systems in medicine should ultimately be judged by clinical significance rather than technical ranking alone [37]. Moreover, real-world performance depends critically on human-AI interaction and implementation context—not merely algorithmic sensitivity [38]. While meta-analyses of RCTs demonstrate improved ADR with CADe systems, the translation of these benefits to routine clinical practice depends on how effectively endoscopists integrate AI alerts into their workflow. Eye-tracking studies have begun to explore how endoscopists and AI interact during procedures [39], but real-world behavioral impacts of AI tools on clinical operators remain insufficiently characterized. A recent study even suggested that routine AI assistance may be associated with a decline in endoscopists’ unassisted detection skills [40]. Our size-dependent efficacy pattern should be interpreted within this framework: AI assistance appears most useful where human perceptual limitations are greatest (diminutive lesions), but platform selection and deployment decisions require prospective evaluation of endoscopist-AI workflow integration, training, and patient-centered outcomes. We therefore caution against using the current exploratory rankings to guide purchasing or clinical prioritization.

Meta-analytic interpretation should distinguish pooled average study outcomes from plausible real-world performance across future clinical settings. Average effects observed across existing trials do not guarantee identical performance in new patient cohorts. Outcomes under real-world conditions could range from measurable benefit to no improvement or inferior detection performance under specific clinical circumstances. This contextual variability largely explains limited interpretive confidence for diminutive-polyp–related findings. For small and large polyps, observations across diverse clinical environments suggest little practical additional benefit provided by AI tools for routine clinical practice. This interpretive distinction is critical for clinical decision-making, consistent with contemporary meta-analysis guidance [41].

Using the GRADE framework, interpretive confidence for primary outcomes was assessed. Confidence was restricted for findings concerning diminutive polyps, driven by serious concerns about cross-study outcome variability, risk of bias (as most studies could not blind operators to the intervention), and indirectness (predominantly Asian populations and mean polyp detection count as a surrogate instead of the preferred ADR). For small and large polyps, confidence remained restricted because of gaps within the available evidence base. A recent meta-analysis of 38 RCTs with GRADE assessment similarly found that evidence certainty ranged from moderate to low for several outcomes due to heterogeneity, inconsistency, and limited data [14]. Therefore, our confidence in the effect estimates is limited, and future research is likely to change these findings.

It is important to distinguish between the average effect observed across aggregated trial data and the expected range of effects across different settings in real-world practice. While the average effect for diminutive polyps suggests a statistically significant benefit, performance in future clinical work could show no benefit or even favor standard colonoscopy in some settings. This distinction is critical for clinical decision-making: the average effect may be modestly positive, but the effect is not consistent across settings, and some populations may derive no benefit or even experience worse outcomes with AI assistance. In contrast, for small and large polyps, both the average effect and the expected range of future effects indicate a consistent null finding with little anticipated variation across settings. This distinction is critical for clinical decision-making, as clinicians must consider not only whether an intervention works on average, but also whether it is likely to work for their specific patients.

The risk of bias assessment using the RoB 2.0 tool revealed that the “deviations from intended interventions” domain was the primary source of bias, with the vast majority of studies rated as high risk or having some concerns. This is an inherent limitation of AI endoscopy trials, as it is not feasible to blind endoscopists and participants to the AI intervention. While unblinded personnel is a methodological constraint rather than a marker of poor study conduct, it does warrant careful interpretation. Unblinded operators might either exert extra effort when AI is active (performance bias) or become overreliant on AI alerts, potentially reducing independent scrutiny [40]. Therefore, observed performance improvements should be interpreted with caution, as part of the apparent benefit could stem from operator behavioral shifts rather than purely AI algorithmic performance. Importantly, the “measurement of the outcome” domain was predominantly low risk, as polyp detection counts were objectively measured and histopathologically confirmed, which strengthens confidence in the outcome data.

Additionally, the predominantly Asian study populations limit generalizability. Baseline ADRs, polyp morphology, and colorectal cancer epidemiology differ between Asian and Western populations, and the cost-effectiveness of AI implementation varies across screening program designs [42]. Our findings should therefore be validated in Western screening cohorts before broad adoption can be recommended.

Several limitations affect interpretation of these findings. First, the absence of head-to-head randomized trials comparing different AI systems necessitates reliance on indirect comparisons through NMA, which carry inherent uncertainty despite methodological safeguards. Second, most constituent studies were conducted in Asian populations, where baseline ADRs, polyp morphology, and colorectal cancer epidemiology differ from Western cohorts [42]; health-economic analyses further indicate that the value proposition for AI-assisted colonoscopy differs across populations and screening-program architectures. Third, the star-shaped network relies entirely on indirect comparisons through standard colonoscopy, with no closed loops to test consistency. The included studies span diverse countries, mixed colonoscopy indications (screening, surveillance, and diagnostic), variable endoscopist experience, and different AI training protocols. These factors are plausible effect modifiers that threaten the transitivity assumption underlying indirect comparisons. Because the network contains only 13 studies and multiple single-study nodes, formal meta-regression to assess these modifiers was not feasible. Readers should therefore view all cross-platform comparisons, including SUCRA rankings, as exploratory and hypothesis-generating rather than evidence of comparative efficacy.

Our NMA findings should not be used to guide clinical decision-making or platform selection in practice. The SUCRA rankings are exploratory and should not be interpreted as evidence of superiority. However, the NMA does offer practical value in a different domain: research prioritization. By synthesizing all available evidence and generating exploratory rankings, the NMA identifies which AI systems show the most promise and therefore warrant evaluation in future head-to-head randomized trials. It also reveals important gaps in the evidence base—for example, which AI systems have not been compared to each other—highlighting where direct evidence is most urgently needed. In this sense, the NMA serves as a tool for guiding the design of future trials, not as a source of practice-changing evidence. We emphasize that direct head-to-head randomized trials are essential before any platform-specific clinical recommendations can be made.

These findings support a stratified approach to AI implementation in colonoscopy practice. For screening colonoscopy where detection of diminutive polyps directly influences surveillance interval assignment, prioritizing high-sensitivity systems such as EndoScreener or comparable CADe platforms appears justified. Conversely, in settings where large lesion detection predominates, such as surveillance of patients with known advanced neoplasia, standard high-definition colonoscopy with rigorous quality assurance may achieve comparable outcomes at lower cost. Health care systems should therefore invest in operator training and procedural quality improvement alongside technology acquisition, recognizing that AI performance is contingent on the endoscopist-AI interaction rather than platform specifications alone.

Future research priorities include head-to-head randomized trials directly comparing multiple AI systems within the same population; validation of size-stratified efficacy in Western screening cohorts; cost-effectiveness analyses incorporating lesion-specific detection rates and downstream surveillance costs [43], and investigation of synergistic combinations between AI and mechanical mucosal exposure devices to address the dual barriers of lesion recognition and visualization [44]. Additionally, real-time AI monitoring of withdrawal quality metrics, such as effective withdrawal time, may offer complementary approaches to optimizing procedure quality independent of detection algorithms themselves [45].

This systematic review delivers 3 distinct novel contributions to the field without overinterpreting indirect cross-AI comparisons: the first size-stratified NMA stratified by polyp diameter, standardized deployment of robust heterogeneity-aware analytical workflows, and stratified GRADE certainty grading to contextualize indirect findings for clinical translation. By demonstrating that the efficacy of CADe varies significantly by lesion size, our stratified analysis reconciles seemingly discordant findings from prior meta-analyses and provides an evidence-based framework for targeted technology deployment. These results suggest that the benefits of AI may be most pronounced for diminutive lesion detection, where human perceptual limitations are greatest, while advanced neoplasia detection may depend more heavily on operator technique and mucosal exposure. This nuance carries immediate implications for endoscopic practice, device selection, and quality improvement prioritization.

Furthermore, by identifying significant heterogeneity in system performance rankings across lesion categories, our findings challenge one-size-fits-all approaches to AI implementation and support the development of lesion-specific performance benchmarks in future technology evaluations. In an era of rapidly proliferating AI devices, such stratified evidence is essential for guiding rational technology adoption, optimizing resource allocation, and ultimately maximizing the population-level impact of colorectal cancer screening programs.

In summary, this review is innovative as the first NMA to combine size-stratified analysis with robust heterogeneity-aware analytical workflows for AI-assisted colonoscopy systems, providing more robust estimates than conventional approaches. It differs from prior reviews by moving beyond overall ADR aggregation to reveal that the AI benefit is primarily driven by diminutive polyp detection, while small and large polyps show minimal or no improvement. This study brings to the field an evidence-based, size-dependent framework that challenges uniform AI deployment and supports lesion-specific performance evaluation. In real-world practice, these findings support a stratified implementation strategy—prioritizing AI for screening populations where diminutive polyp detection directly influences surveillance intervals, while recognizing that for large lesion detection, standard high-definition colonoscopy with rigorous quality assurance may achieve comparable outcomes at lower cost. Direct head-to-head randomized trials comparing multiple AI systems in diverse populations, using patient-centered outcomes, are urgently needed to provide high-certainty evidence.

## Appendix

Multimedia Appendix 1 PRISMA 2020 for abstract checklist.

Multimedia Appendix 2 PRISMA 2020 expanded checklist.

Multimedia Appendix 3 PRISMA-S checklist.

Multimedia Appendix 4 Complete search strategies and search results.

Multimedia Appendix 5 GRADE assessment of outcome measures.

Multimedia Appendix 6 Extracted data.

Multimedia Appendix 7 R code.

Multimedia Appendix 8 Supplementary figures.

## Abbreviations

ADR: adenoma detection rate
CADe: computer-aided detection
GRADE: Grading of Recommendations Assessment, Development, and Evaluation
HKSJ: Hartung-Knapp-Sidik-Jonkman
NMA: network meta-analysis
PDR: polyp detection rate
PI: prediction interval
PRISMA: Preferred Reporting Items for Systematic Reviews and Meta-Analyses
PROSPERO: International Prospective Register of Systematic Reviews
RCT: randomized controlled trial
REML: restricted maximum likelihood
RoB: Risk of Bias
SMD: standardized mean difference
SUCRA: surface under the cumulative ranking curve

## Data Availability

The authors would provide the raw data that supports the conclusions of this article, without any undue restrictions.
