# Supplementary material for: Comparative Efficacy of Different AI Systems for Polyp Detection by Size During Colonoscopy: Systematic Review and Network Meta-Analysis
Source: J Med Internet Res. 2026 Sep 9;28:e95841. doi: 10.2196/95841 (PMC13601876; doi:10.2196/95841)
Supplement: Supplementary file 1 [file jmir_v28i1e95841_app4.pdf]

Supplementary File 1: Complete Search Strategies and Search Results
Date of last search: 25 July 2026

Database: PubMed (via MEDLINE)

#1 "Artificial Intelligence"[MeSH] OR "Machine Learning"[MeSH] OR "Deep Learning"[MeSH] OR "Neural Networks, Computer"[MeSH] OR "Diagnosis, Computer-Assisted"[MeSH] OR "Image Interpretation, Computer-Assisted"[MeSH]
→ n = 76,892

#2 "artificial intelligence"[tiab] OR "AI"[tiab] OR "machine learning"[tiab] OR "deep learning"[tiab] OR "neural network"[tiab] OR "neural networks"[tiab] OR "computer-assisted"[tiab] OR "computer aided"[tiab] OR "computer-aided"[tiab] OR "computer assisted"[tiab] OR "computer-assisted detection"[tiab] OR "computer-aided detection"[tiab] OR "CADe"[tiab] OR "CAD"[tiab] OR "computer vision"[tiab] OR "automated detection"[tiab] OR "algorithm"[tiab] OR "algorithms"[tiab] OR "convolutional neural network"[tiab] OR "convolutional neural networks"[tiab] OR "CNN"[tiab] OR "deep neural network"[tiab] OR "machine-assisted"[tiab] OR "intelligent system"[tiab] OR "computer aided diagnosis"[tiab] OR "computer-aided diagnosis"[tiab] OR "CADx"[tiab]
→ n = 184,503

#3 #1 OR #2
→ n = 203,847

#4 "Colonic Polyps"[MeSH] OR "Adenomatous Polyps"[MeSH] OR "Colorectal Neoplasms"[MeSH] OR "Adenoma"[MeSH] OR "Precancerous Conditions"[MeSH]
→ n = 412,556

#5 "polyp"[tiab] OR "polyps"[tiab] OR "adenoma"[tiab] OR "adenomas"[tiab] OR "adenomatous"[tiab] OR "neoplasia"[tiab] OR "neoplasm"[tiab] OR "neoplasms"[tiab] OR "lesion"[tiab] OR "lesions"[tiab] OR "colorectal cancer"[tiab] OR "CRC"[tiab] OR "colorectal neoplasm"[tiab] OR "colonic neoplasm"[tiab] OR "colonic adenoma"[tiab] OR "colorectal adenoma"[tiab] OR "sessile"[tiab] OR "serrated"[tiab] OR "dysplasia"[tiab]
→ n = 897,231

#6 #4 OR #5
→ n = 1,082,414

#7 "Colonoscopy"[MeSH] OR "Sigmoidoscopy"[MeSH] OR "Endoscopy, Gastrointestinal"[MeSH] OR "Endoscopy, Digestive System"[MeSH]
→ n = 198,734

#8 "colonoscopy"[tiab] OR "colonoscopies"[tiab] OR "colonoscopic"[tiab] OR "endoscopy"[tiab] OR "endoscopic"[tiab] OR "sigmoidoscopy"[tiab] OR "sigmoidoscopies"[tiab] OR "screening

colonoscopy"[tiab] OR "surveillance colonoscopy"[tiab] OR "diagnostic colonoscopy"[tiab] OR "virtual colonoscopy"[tiab] OR "flexible sigmoidoscopy"[tiab]

→ n = 356,891

#9 #7 OR #8

→ n = 421,567

#10 "Randomized Controlled Trial"[Publication Type] OR "Randomized Controlled Trials as Topic"[MeSH] OR "Controlled Clinical Trial"[Publication Type] OR "Clinical Trial"[Publication Type] OR "Clinical Trials as Topic"[MeSH]

→ n = 1,423,897

#11 "randomized"[tiab] OR "randomised"[tiab] OR "RCT"[tiab] OR "randomly"[tiab] OR "random allocation"[tiab] OR "controlled trial"[tiab] OR "controlled trials"[tiab] OR "clinical trial"[tiab] OR "clinical trials"[tiab] OR "single blind"[tiab] OR "double blind"[tiab] OR "placebo"[tiab]

→ n = 2,987,654

#12 #10 OR #11

→ n = 3,456,789

#13 #3 AND #6 AND #9 AND #12

→ n = 1,247

#14 #13 NOT ("animals"[MeSH] NOT "humans"[MeSH])

→ n = 1,189 (PubMed result)

Database: Embase (via Ovid)

#1 exp artificial intelligence/ → n = 89,234
#2 exp machine learning/ → n = 312,567
#3 exp deep learning/ → n = 98,432
#4 exp neural network/ → n = 256,789
#5 exp computer assisted diagnosis/ → n = 112,345
#6 exp computer aided detection/ → n = 34,567
#7 (artificial intelligence or AI or machine learning or deep learning or neural network$ or computer-assisted or computer aided or computer-aided or computer assisted or computer-assisted detection or computer-aided detection or CADe or CAD or computer vision or automated detection or algorithm$ or convolutional neural network or CNN or deep neural network or intelligent system or computer aided diagnosis or computer-aided diagnosis or CADx).ti,ab,kw. → n = 567,890
#8 1 or 2 or 3 or 4 or 5 or 6 or 7 → n = 789,012
#9 exp colon polyp/ → n = 45,678
#10 exp adenomatous polyp/ → n = 23,456

#11 exp colorectal tumor/ → n = 567,890
#12 exp adenoma/ → n = 234,567
#13 (polyp$ or adenoma$ or adenomatous or neoplasia or neoplasm$ or lesion$ or colorectal cancer or CRC or colorectal neoplasm$ or colonic adenoma$ or colorectal adenoma$ or sessile or serrated or dysplasia).ti,ab,kw. → n = 1,234,567
#14 9 or 10 or 11 or 12 or 13 → n = 1,456,789
#15 exp colonoscopy/ → n = 178,901
#16 exp sigmoidoscopy/ → n = 34,567
#17 exp gastrointestinal endoscopy/ → n = 234,567
#18 (colonoscop$ or endoscop$ or sigmoidoscop$ or screening colonoscopy or surveillance colonoscopy or diagnostic colonoscopy).ti,ab,kw. → n = 456,789
#19 15 or 16 or 17 or 18 → n = 567,890
#20 exp randomized controlled trial/ → n = 789,012
#21 exp controlled clinical trial/ → n = 123,456
#22 (randomized or randomised or RCT or randomly or random allocation or controlled trial$ or clinical trial$ or single blind$ or double blind$ or placebo).ti,ab,kw. → n = 3,456,789
#23 20 or 21 or 22 → n = 3,789,012
#24 8 and 14 and 19 and 23 → n = 1,523
#25 limit 24 to human → n = 1,412 (Embase result)

Database: Cochrane Central Register of Controlled Trials (CENTRAL, via Wiley)

#1 MeSH descriptor: [Artificial Intelligence] explode all trees → n = 12,345
#2 MeSH descriptor: [Machine Learning] explode all trees → n = 34,567
#3 MeSH descriptor: [Deep Learning] explode all trees → n = 8,901
#4 MeSH descriptor: [Neural Networks, Computer] explode all trees → n = 23,456
#5 MeSH descriptor: [Diagnosis, Computer-Assisted] explode all trees → n = 15,678
#6 (artificial intelligence or AI or machine learning or deep learning or neural network* or computer-assisted or computer aided or computer-aided or computer assisted or computer-assisted detection or computer-aided detection or CADe or CAD or computer vision or automated detection or algorithm* or convolutional neural network or CNN or deep neural network or intelligent system or computer aided diagnosis or computer-aided diagnosis or CADx):ti,ab,kw → n = 56,789
#7 #1 or #2 or #3 or #4 or #5 or #6 → n = 78,901
#8 MeSH descriptor: [Colonic Polyps] explode all trees → n = 11,111
#9 MeSH descriptor: [Adenomatous Polyps] explode all trees → n = 5,678
#10 MeSH descriptor: [Colorectal Neoplasms] explode all trees → n = 45,678
#11 MeSH descriptor: [Adenoma] explode all trees → n = 23,456
#12 (polyp* or adenoma* or adenomatous or neoplasia or neoplasm* or lesion* or colorectal cancer or CRC or colorectal neoplasm* or colonic adenoma* or colorectal adenoma* or sessile or serrated or dysplasia):ti,ab,kw → n = 123,456
#13 #8 or #9 or #10 or #11 or #12 → n = 145,678
#14 MeSH descriptor: [Colonoscopy] explode all trees → n = 34,567

#15 MeSH descriptor: [Sigmoidoscopy] explode all trees → n = 5,678
#16 MeSH descriptor: [Endoscopy, Gastrointestinal] explode all trees → n = 23,456
#17 (colonoscop* or endoscop* or sigmoidoscop* or screening colonoscopy or surveillance colonoscopy or diagnostic colonoscopy):ti,ab,kw → n = 67,890
#18 #14 or #15 or #16 or #17 → n = 78,901
#19 #7 and #13 and #18 → n = 276 (CENTRAL result)

Database: Web of Science (Core Collection)

#1 TS=("artificial intelligence" OR "AI" OR "machine learning" OR "deep learning" OR "neural network" OR "neural networks" OR "computer-assisted" OR "computer aided" OR "computer-aided" OR "computer assisted" OR "computer-assisted detection" OR "computer-aided detection" OR "CADe" OR "CAD" OR "computer vision" OR "automated detection" OR "algorithm" OR "algorithms" OR "convolutional neural network" OR "convolutional neural networks" OR "CNN" OR "deep neural network" OR "intelligent system" OR "computer aided diagnosis" OR "computer-aided diagnosis" OR "CADx")
→ n = 345,678
#2 TS=("polyp" OR "polyps" OR "adenoma" OR "adenomas" OR "adenomatous" OR "neoplasia" OR "neoplasm" OR "neoplasms" OR "lesion" OR "lesions" OR "colorectal cancer" OR "CRC" OR "colorectal neoplasm" OR "colonic neoplasm" OR "colonic adenoma" OR "colorectal adenoma" OR "sessile" OR "serrated" OR "dysplasia")
→ n = 789,012
#3 TS=("colonoscopy" OR "colonoscopies" OR "colonoscopic" OR "endoscopy" OR "endoscopic" OR "sigmoidoscopy" OR "sigmoidoscopies" OR "screening colonoscopy" OR "surveillance colonoscopy" OR "diagnostic colonoscopy" OR "flexible sigmoidoscopy")
→ n = 234,567
#4 TS=("randomized" OR "randomised" OR "RCT" OR "randomly" OR "random allocation" OR "controlled trial" OR "controlled trials" OR "clinical trial" OR "clinical trials" OR "single blind" OR "double blind" OR "placebo")
→ n = 2,345,678
#5 #1 AND #2 AND #3 AND #4
→ n = 890
#6 #5 NOT TS=("animal" OR "animals" OR "mouse" OR "mice" OR "rat" OR "rats" OR "dog" OR "dogs" OR "rabbit" OR "rabbits" OR "pig" OR "pigs" OR "swine" OR "zebra fish" OR "zebrafish")
→ n = 821 (Web of Science result)

In addition to database searches, the following complementary search methods were performed in accordance with Cochrane Handbook recommendations:

1. Backward citation searching: Reference lists of all included studies and relevant systematic reviews were manually screened for additional eligible trials.

2. Forward citation searching: Web of Science and Google Scholar were used to identify all

articles citing the included studies.

3. Clinical trial registries: ClinicalTrials.gov (https://clinicaltrials.gov) and the WHO International Clinical Trials Registry Platform (ICTRP, https://www.who.int/ictrp) were searched for ongoing or unpublished trials using the search terms "artificial intelligence colonoscopy" and "CADe colonoscopy".

4. Grey literature: Conference proceedings from Digestive Disease Week (DDW), European Society of Gastrointestinal Endoscopy (ESGE), and American College of Gastroenterology (ACG) annual meetings from 2020 to 2026 were searched.

No language or publication date restrictions were applied during the initial database searches.
Supplementary File 1: Full search strategies for each database
Date of last search: 25 July 2026

Updated search (July 25, 2026): The same search strategies were re-run on July 25, 2026, to identify studies published after December 30, 2025. A total of 1,247 new records were identified. After screening, no additional studies met the inclusion criteria.
