# Supplementary material for: Comparative Efficacy of Different AI Systems for Polyp Detection by Size During Colonoscopy: Systematic Review and Network Meta-Analysis
Source: J Med Internet Res. 2026 Sep 9;28:e95841. doi: 10.2196/95841 (PMC13601876; doi:10.2196/95841)
Supplement: Supplementary file 2 [file jmir_v28i1e95841_app5.pdf]

Certainty assessment							№ of patients		Effect	Certainty	Importance
No of studies	Study design	Risk of bias	Inconsistency	Indirectness	Imprecision	Other considerations	Intervention	Control	Effect size (95% CI)		
**Mean number of diminutive polyps detected (≤5 mm)**											
13	randomised trials	serious[a]	very serious[b]	not serious	not serious	none	4750	4734	SMD **0.21** (0.07 to 0.35)	Very low[a,b]	Critical
**Mean number of small polyps detected (6-9 mm)**											
13	randomised trials	serious[a]	not serious	not serious	serious[c]	none	4750	4734	SMD **0.02** (-0.02 to 0.06)	Low[a,c]	Critical
**Mean number of large polyps detected (≥10 mm)**											
13	randomised trials	serious[a]	not serious	not serious	serious[d]	none	4750	4734	SMD **0.01** (0.00 to 0.02)	Low[a,d]	Critical

CI: confidence interval; **SMD:** standardised mean difference

Explanations

a. Most trials lacked endoscopist blinding; partial inadequate allocation concealment.
b. I^2 = 86.6%, 95% prediction interval crosses null, large between- study heterogeneity.
c. Effect estimate close to null; wide confidence interval crossing no- effect.
d. Effect estimate close to the null; trivial clinical magnitude.
