# Supplementary material for: Comparative Efficacy of Different AI Systems for Polyp Detection by Size During Colonoscopy: Systematic Review and Network Meta-Analysis
Source: J Med Internet Res. 2026 Sep 9;28:e95841. doi: 10.2196/95841 (PMC13601876; doi:10.2196/95841)
Supplement: Supplementary file 3 [file jmir_v28i1e95841_app7.pdf]

Forest

```
library(ggplot2)
library(metafor)

fig_dir <- "E:/JMI-R/minor"
if (!dir.exists(fig_dir)) dir.create(fig_dir, recursive = TRUE)

data_d <- data.frame(
  AI_system = c("Olympus", "EndoScreener", "CAD EYE", "CAD EYE",
                "EndoScreener", "GI Genius", "Wision AI", "Tianjin YuJin",
                "EndoAngel", "EndoScreener", "AI-assisted", "AI-assisted",
                "EndoAngel"),
  effect = c(0.5834053, 0.3618555, -0.2392380, 0.06171033, -0.07602574,
             0.2467532, 0.3949648, 0.1331019, 0.1313268, 0.5381711,
             0.1250550, 0.6108899, 0.1184229),
  se = c(0.2264017, 0.09298263, 0.2543248, 0.03695299, 0.1003289,
         1.620795, 0.1200102, 0.8739501, 0.04247939, 0.1635080,
         0.03984764, 0.1856176, 0.04102131)
)

data_s <- data.frame(
  AI_system = data_d$AI_system,
  effect = c(0.10119375, 0.02920225, -0.2558872, 0.1168246, 0.1273532,
             0.01332654, 0.04519786, 0.08217593, 0.03022250, 0.02410573,
             -0.02060486, 0.04766972, 0.005294806),
  se = c(0.03927019, 0.02592451, 0.5834074, 0.06889103, 0.1680642,
         0.01261978, 0.02275316, 0.2643387, 0.02279803, 0.02573830,
         0.00649005, 0.02344013, 0.01242441)
)

data_l <- data.frame(
  AI_system = data_d$AI_system,
  effect = c(0.08631116, -0.00028528, -0.08099595, 0.01960238, 0.07079646,
             0.08242085, 0.01199462, 0.11631944, 0.01131795, -0.00488396,
             0.00414975, 0.01241906, 0.02238993),
  se = c(0.03349471, 0.01137963, 0.08556971, 0.10092010, 0.09342800,
         0.06777697, 0.00973662, 0.08328203, 0.01119352, 0.00949493,
         0.00665625, 0.00992834, 0.01248718)
)

draw_forest <- function(dat, title, grade, filename, x_limits) {

  res <- rma(yi = effect, sei = se, data = dat, method = "REML", test = "knha")
```

```r
dat$lower <- dat$effect - 1.96 * dat$se
dat$upper <- dat$effect + 1.96 * dat$se
dat$weight <- 1 / dat$se^2
dat$weight_norm <- dat$weight / max(dat$weight) * 3

pi_lb <- res$b[1] - qt(0.975, df = res$k - 2) * sqrt(res$tau2 + res$se[1]^2)
pi_ub <- res$b[1] + qt(0.975, df = res$k - 2) * sqrt(res$tau2 + res$se[1]^2)

dat <- dat[order(dat$effect, decreasing = TRUE), ]
dat$order <- 1:nrow(dat)
n <- nrow(dat)

  diamond_y <- -1.0
overall_y <- -1.8          pi_y        <- -2.8        pi_label    <- -3.4
het_y       <- -4.2          grade_y     <- -5.0          k_y          <- -5.7
p <- ggplot(dat) +

     geom_vline(xintercept = 0, linetype = "dashed", color = "gray50", size = 0.7) +

     geom_segment(aes(x = pi_lb, xend = pi_ub, y = pi_y, yend = pi_y),
                  color = "red", size = 1.2, linetype = "dashed") +
  geom_point(aes(x = res$b[1], y = pi_y), color = "red", size = 2.5, shape = 18) +

      geom_errorbarh(aes(xmin = lower, xmax = upper, y = order),
                     height = 0.15, color = "black", size = 0.6) +

      geom_point(aes(x = effect, y = order, size = weight_norm),
                 shape = 15, color = "black", fill = "gray30") +
  scale_size_continuous(range = c(1.2, 5), guide = "none") +

      geom_polygon(data = data.frame(
    x = c(res$ci.lb, res$b[1], res$ci.ub, res$b[1]),
    y = c(diamond_y - 0.4, diamond_y, diamond_y - 0.4, diamond_y - 0.8)
  ), aes(x = x, y = y), fill = "blue", color = "blue", alpha = 0.3) +

    geom_text(aes(x = x_limits[1] - 0.15, y = order, label = AI_system),
           hjust = 1, vjust = 0.5, size = 4.5, fontface = "bold") +

    geom_text(aes(x = x_limits[2] + 0.25, y = order,
                  label = paste0(sprintf("%.2f", effect), " [",
                                 sprintf("%.2f", lower), ", ",
                                 sprintf("%.2f", upper), "]")),
           hjust = 0, vjust = 0.5, size = 3.8) +
```

```
  geom_text(aes(x = x_limits[2] - 0.4, y = order,
                label = paste0(sprintf("%.1f", weight / sum(weight) * 100), "%")),
            hjust = 1, vjust = 0.5, size = 3.5, color = "gray30") +

 geom_text(aes(x = res$b[1], y = overall_y,
                label = paste0("Overall (HKSJ): ",
                               sprintf("%.2f", res$b[1]), " [",
                               sprintf("%.2f", res$ci.lb), ", ",
                               sprintf("%.2f", res$ci.ub), "]")),
            hjust = 0.5, vjust = 1, size = 5.0,
            color = "#1E40AF", fontface = "bold") +

 geom_text(aes(x = x_limits[1] - 0.15, y = pi_label,
                label = paste0("95% Prediction Interval: ",
                               sprintf("%.2f", pi_lb), " to ", sprintf("%.2f", pi_ub))),
            hjust = 0, vjust = 0.5, size = 3.6, color = "red") +

 geom_text(aes(x = x_limits[1] - 0.15, y = het_y,
                label = paste0("Heterogeneity: I²   = ", round(res$I2, 1),
                               "%   |   τ ²  = ", sprintf("%.4f", res$tau2),
                               "   |   Q test p = ", sprintf("%.4f", res$QEp))),
            hjust = 0, vjust = 0.5, size = 3.6) +

geom_text(aes(x = x_limits[1] - 0.15, y = grade_y,
                label = paste0("GRADE Certainty: ", grade)),
            hjust = 0, vjust = 0.5, size = 4.5, color = "blue", fontface = "bold") +

 geom_text(aes(x = x_limits[1] - 0.15, y = k_y,
                label = paste0("Number of studies: k = ", n)),
            hjust = 0, vjust = 0.5, size = 3.6) +

  geom_text(aes(x = x_limits[1] - 0.15, y = n + 1.2, label = "Study"),
            hjust = 1, vjust = 0.5, size = 4.5, fontface = "bold") +
geom_text(aes(x = (x_limits[1] + x_limits[2]) / 2, y = n + 1.2, label = "SMD [95% CI]"),
              hjust = 0.5, vjust = 0.5, size = 4.5, fontface = "bold") +
geom_text(aes(x = x_limits[2] - 0.4, y = n + 1.2, label = "Weight"),
              hjust = 1, vjust = 0.5, size = 4.5, fontface = "bold") +
geom_text(aes(x = x_limits[2] + 0.25, y = n + 1.2, label = "SMD [95% CI]"),
              hjust = 0, vjust = 0.5, size = 4.5, fontface = "bold") +

 scale_x_continuous(limits = c(x_limits[1] - 0.5, x_limits[2] + 0.6),
                     breaks = seq(floor(x_limits[1]), ceiling(x_limits[2]), by = 1)) +
scale_y_reverse(breaks = NULL, expand = expansion(mult = c(0.1, 0.30))) +
```

```
    labs(title = title,
         x = "Favours Standard    ←    |    →    Favours AI",
         y = NULL) +

    theme_minimal(base_size = 12) +
    theme(
      plot.title = element_text(size = 16, face = "bold", hjust = 0.5, margin = margin(b = 15)),
      axis.title.x = element_text(size = 11, face = "bold", margin = margin(t = 15)),
      axis.text.x = element_text(size = 10),
      axis.text.y = element_blank(),
      panel.grid.major = element_blank(),
      panel.grid.minor = element_blank(),
      plot.margin = margin(10, 20, 10, 20)
    )

  ggsave(filename, plot = p, width = 11, height = 11, dpi = 300, bg = "white")
  cat("已生成：", filename, "\n")
  print(p)
}

draw_forest(data_d, "Diminutive Polyps (≤5 mm)", "Very Low",
            file.path(fig_dir, "Forest_Plot_Diminutive.png"), x_limits = c(-2.2, 2.2))
draw_forest(data_s, "Small Polyps (6-9 mm)", "Low",
            file.path(fig_dir, "Forest_Plot_Small.png"), x_limits = c(-0.6, 0.6))
draw_forest(data_l, "Large Polyps (≥10 mm)", "Low",
            file.path(fig_dir, "Forest_Plot_Large.png"), x_limits = c(-0.2, 0.2))

data_d_sens <- data_d[data_d$AI_system != "Tianjin YuJin", ]
data_s_sens <- data_s[data_s$AI_system != "Tianjin YuJin", ]
data_l_sens <- data_l[data_l$AI_system != "Tianjin YuJin", ]

draw_forest(data_d_sens, "Diminutive Polyps (≤5 mm) - Sensitivity (excl. Tianjin YuJin)", "Very
Low",
            file.path(fig_dir, "Forest_Sensitivity_Diminutive.png"), x_limits = c(-2.2, 2.2))
draw_forest(data_s_sens, "Small Polyps (6-9 mm) - Sensitivity (excl. Tianjin YuJin)", "Low",
            file.path(fig_dir, "Forest_Sensitivity_Small.png"), x_limits = c(-0.6, 0.6))
draw_forest(data_l_sens, "Large Polyps (≥10 mm) - Sensitivity (excl. Tianjin YuJin)", "Low",
            file.path(fig_dir, "Forest_Sensitivity_Large.png"), x_limits = c(-0.2, 0.2))

cat("\n========== 全部完成 ==========\n")
cat("所有图片已保存至：", fig_dir, "\n")
```

Funnel

```
if (!require(metafor)) install.packages("metafor")
library(metafor)

manual_funnel <- function(data, title, filename) {
    res <- rma(yi = effect, sei = se, data = data, method = "REML", test = "knha")

  eff <- data$effect
  se <- data$se
  inv_se <- 1 / se     #
  xlim <- c(min(eff) - 0.2, max(eff) + 0.2)
  ylim <- c(0, max(inv_se) * 1.05)
  png(filename, width = 6, height = 6, units = "in", res = 300)

  plot(NA, xlim = xlim, ylim = ylim,
       xlab = "Standardized Mean Difference",
       ylab = "1 / Standard Error (precision)",
       main = title)

  se_range <- seq(min(se), max(se), length.out = 100)
  inv_se_range <- 1 / se_range
  lower_bound <- res$b[1] - 1.96 * se_range
  upper_bound <- res$b[1] + 1.96 * se_range
  lines(lower_bound, inv_se_range, col = "gray50", lty = 2)
  lines(upper_bound, inv_se_range, col = "gray50", lty = 2)
  abline(v = res$b[1], col = "gray30", lty = 1)

  points(eff, inv_se, pch = 21, bg = "gray60", cex = 1.2)

  egger <- regtest(res, model = "rma")
  p_val <- round(egger$pval, 3)
  legend("bottomright", legend = paste("Egger's test p =", p_val),
         bty = "n", cex = 0.8, text.col = "blue")

  dev.off()
  cat("已保存：", filename, " (p =", p_val, ")\n")
}

manual_funnel(data_d, "Diminutive polyps (≤5 mm)", "Fig7A_Funnel_Diminutive_manual.png")
manual_funnel(data_s, "Small polyps (6-9 mm)",      "Fig7B_Funnel_Small_manual.png")
manual_funnel(data_l, "Large polyps (≥10 mm)",      "Fig7C_Funnel_Large_manual.png")
```

League

```
if (!require(metafor)) install.packages("metafor")
if (!require(gridExtra)) install.packages("gridExtra")
if (!require(grid)) install.packages("grid")
library(metafor)
library(gridExtra)
library(grid)

data_d <- data.frame(
  AI_system = c("Olympus","EndoScreener","CAD EYE","CAD EYE","EndoScreener",
                "GI Genius","Wision AI","Tianjin YuJin","EndoAngel","EndoScreener",
                "AI-assisted","AI-assisted","EndoAngel"),
  effect = c(0.5834053,0.3618555,-0.2392380,0.06171033,-0.07602574,
             0.2467532,0.3949648,0.1331019,0.1313268,0.5381711,
             0.1250550,0.6108899,0.1184229),
  se = c(0.2264017,0.09298263,0.2543248,0.03695299,0.1003289,
         1.620795,0.1200102,0.8739501,0.04247939,0.1635080,
         0.03984764,0.1856176,0.04102131),
  n = c(238,484,113,207,113,308,522,64,268,393,1177,508,355)
)

data_s <- data.frame(
  AI_system = data_d$AI_system,
  effect = c(0.10119375,0.02920225,-0.2558872,0.1168246,0.1273532,
             0.01332654,0.04519786,0.08217593,0.03022250,0.02410573,
             -0.02060486,0.04766972,0.005294806),
  se = c(0.03927019,0.02592451,0.5834074,0.06889103,0.1680642,
         0.01261978,0.02275316,0.2643387,0.02279803,0.02573830,
         0.00649005,0.02344013,0.01242441),
  n = data_d$n
)

data_l <- data.frame(
  AI_system = data_d$AI_system,
  effect = c(0.08631116,-0.00028528,-0.08099595,0.01960238,0.07079646,
             0.08242085,0.01199462,0.11631944,0.01131795,-0.00488396,
             0.00414975,0.01241906,0.02238993),
  se = c(0.03349471,0.01137963,0.08556971,0.10092010,0.09342800,
         0.06777697,0.00973662,0.08328203,0.01119352,0.00949493,
         0.00665625,0.00992834,0.01248718),
  n = data_d$n
)

draw_league <- function(data, size_name, filename, width_inch = 18) {
```

```
agg <- aggregate(cbind(effect, se) ~ AI_system, data, mean)
trt <- agg$AI_system
k <- length(trt)
mat <- matrix("", k, k)
rownames(mat) <- trt
colnames(mat) <- trt
for (i in 1:k) {
  for (j in 1:k) {
    if (i <= j) next
    diff <- agg$effect[i] - agg$effect[j]
    se_diff <- sqrt(agg$se[i]^2 + agg$se[j]^2)
    lower <- diff - 1.96 * se_diff
    upper <- diff + 1.96 * se_diff
    mat[i, j] <- sprintf("%.2f (%.2f, %.2f)", diff, lower, upper)
  }
}

df <- as.data.frame(mat, stringsAsFactors = FALSE)
df <- cbind(Treatment = rownames(df), df)
colnames(df) <- c("Treatment", trt)

my_theme <- ttheme_default(
  core = list(fg_params = list(hjust = 0.5, x = 0.5, cex = 0.6),
              bg_params = list(fill = c("white", "gray95"))),
  colhead = list(fg_params = list(hjust = 0.5, x = 0.5, fontface = "bold", cex = 0.7),
                 bg_params = list(fill = "lightgray")),
  rownames = list(fg_params = list(hjust = 0.5, x = 0.5, fontface = "bold", cex = 0.6),
                  bg_params = list(fill = "lightgray"))
)

tbl <- tableGrob(df, rows = NULL, theme = my_theme)

title <- textGrob(paste("Indirect comparisons for", size_name, "polyps"),
                  gp = gpar(fontsize = 12, fontface = "bold"))
subtitle <- textGrob("SMD (95% CI); row intervention minus column intervention",
                     gp = gpar(fontsize = 9))
footnote <- textGrob("Note: All comparisons are indirect via standard colonoscopy. No head-to-head trials exist.",
                     gp = gpar(fontsize = 8, col = "gray30"), just = "left")

height_inch <- 0.5 * (nrow(df) + 4)

png(filename, width = width_inch, height = height_inch, units = "in", res = 300)
grid.arrange(title, subtitle, tbl, footnote,
```

```
                         nrow = 4,
                         heights = c(0.6, 0.4, nrow(df) * 0.5, 0.6))
    dev.off()
    cat("已保存：", filename, "\n")
}

draw_league(data_d, "Diminutive (≤5 mm)", "League_Diminutive.png", width_inch = 18)
draw_league(data_s, "Small (6-9 mm)",          "League_Small.png",          width_inch = 18)
draw_league(data_l, "Large (≥10 mm)",          "League_Large.png",          width_inch = 18)

cat("全部联赛表生成完毕！\n")
```
