# Supplementary material for: Comparative Efficacy of Different AI Systems for Polyp Detection by Size During Colonoscopy: Systematic Review and Network Meta-Analysis
Source: J Med Internet Res. 2026 Sep 9;28:e95841. doi: 10.2196/95841 (PMC13601876; doi:10.2196/95841)
Supplement: Supplementary file 5 [file jmir_v28i1e95841_app2.docx]

PRISMA 2020 expanded checklist

Note: This expanded checklist details elements recommended for reporting for each item in the PRISMA 2020 statement. Non-italicized elements are considered ‘essential’ and should be reported in the main report or as supplementary material for all systematic reviews (except for those preceded by “If…”, which should only be reported where applicable). Elements written in italics are‘additional’, and while not essential, provide supplementary information that may enhance the completeness and usability of systematic review reports. Note that elements presented here are an abridged version of those presented in the explanation and elaboration paper (BMJ 2021;372:n160), with references and some examples removed. Consulting the explanation and elaboration paper is recommended if further clarity or information is required.

| Section/Topic | Item | Checklist item | Location reported |
| --- | --- | --- | --- |
| TITLE | 1 | Identify the report as a systematic review. | Title page |
| ABSTRACT | 2 | See PRISMA 2020 for Abstracts checklist. | Abstract |
| INTRODUCTION |  |  |  |
| Rationale | 3 | Describe rationale for review in context of existing knowledge. | Introduction, para 1‑4 |
| Objectives | 4 | Provide explicit statement of objectives or questions. | Introduction, last para |
| METHODS |  |  |  |
| Eligibility criteria | 5 | Specify inclusion/exclusion criteria and how studies were grouped. | Methods – Eligibility criteria |
| Information sources | 6 | Specify all databases, registers, etc., and date last searched. | Methods – Search strategies |
| Search strategy | 7 | Present full search strategies for all databases. | Supplementary File 1 |
| Selection process | 8 | Specify methods for deciding eligibility, number of reviewers, automation. | Methods – Selection process |
| Data collection process | 9 | Specify methods for data collection, number of reviewers, independence. | Methods – Data extraction |
| Data items | 10a | List and define all outcomes for which data were sought. | Methods – Data items |
| 10b | List and define all other variables. | Methods – Data items |  |
| Study risk of bias assessment | 11 | Specify methods for risk of bias assessment (RoB 1.0). | Methods – Risk of bias assessment |
| Effect measures | 12 | Specify for each outcome the effect measure(s). | Methods – Effect measures |
| Synthesis methods | 13a | Describe processes to decide which studies eligible for each synthesis. | Methods – Synthesis methods |
| 13b | Describe methods to prepare data for synthesis. | Methods – Synthesis methods |  |
| 13c | Describe methods to tabulate or visually display results. | Methods – Synthesis methods |  |
| 13d | Describe methods to synthesise results, model, heterogeneity measures, software. | Methods – Synthesis methods |  |
| 13e | Describe methods to explore possible causes of heterogeneity. | Methods – Synthesis methods |  |
| 13f | Describe sensitivity analyses. | Methods – Synthesis methods |  |
| Reporting bias assessment | 14 | Describe methods to assess risk of bias due to missing results (reporting biases). | Methods – Reporting bias assessment |
| Certainty assessment | 15 | Describe methods to assess certainty (GRADE). | Methods – Certainty assessment |
| RESULTS |  |  |  |
| Study selection | 16a | Describe results of search and selection, ideally with flow diagram. | Results – Study selection; Figure 1 |
| 16b | Cite excluded studies and explain reasons. | Results – (stated none) |  |
| Study characteristics | 17 | Cite each included study and present its characteristics. | Table 1 |
| Risk of bias in studies | 18 | Present assessments of risk of bias for each included study. | Figure 10, Figure 16; Supplementary Table S3 |
| Results of individual studies | 19 | For all outcomes, present summary statistics and effect estimates per study. | Forest plots (Figures 4,5,7,11,14,15); Table 1 |
| Results of syntheses | 20a | Summarise characteristics and risk of bias among contributing studies. | Results – Size‑stratified analysis |
| 20b | Present results of all statistical syntheses. | Results – Size‑stratified analysis; Figures 3‑8, 14‑15 |  |
| 20c | Present results of investigations of heterogeneity. | Results – Size‑stratified analysis (I², PI) |  |
| 20d | Present results of sensitivity analyses. | Results – Sensitivity analysis; Figures S1‑S3 |  |
| Reporting biases | 21 | Present assessments of risk of bias due to missing results. | Results – Small‑study effects; Figure 9, Figure 17 |
| Certainty of evidence | 22 | Present assessments of certainty (GRADE). | Results – GRADE certainty assessment; Supplementary Table S5 |
| DISCUSSION |  |  |  |
| 23a | Provide general interpretation. | Discussion (first para) |  |
| 23b | Discuss limitations of evidence. | Discussion (Limitations) |  |
| 23c | Discuss limitations of review processes. | Discussion (Limitations) |  |
| 23d | Discuss implications for practice, policy, and future research. | Discussion (Implications) |  |
| OTHER INFORMATION |  |  |  |
| Registration and protocol | 24a | Provide registration information. | Methods – Registration |
| 24b | Indicate where protocol can be accessed. | Methods – Registration |  |
| 24c | Describe any amendments. | Methods – Registration (none) |  |
| Support | 25 | Describe sources of financial or non‑financial support. | Funding statement |
| Competing interests | 26 | Declare competing interests. | Conflict of Interest |
| Availability of data, code, other materials | 27 | Report which materials are publicly available and where. | Methods – Certainty assessment (Data and code availability) |
