# Supplementary material for: Comparative Efficacy of Different AI Systems for Polyp Detection by Size During Colonoscopy: Systematic Review and Network Meta-Analysis
Source: J Med Internet Res. 2026 Sep 9;28:e95841. doi: 10.2196/95841 (PMC13601876; doi:10.2196/95841)
Supplement: Supplementary file 7 [file jmir_v28i1e95841_app6.docx]

|  | Diminutive (s5 mm) |  |  |  |  |
| --- | --- | --- | --- | --- | --- |
| id | study id | t | mean | sd | n |
| 1 | Thomas Ka-Luen Lui 2024 | Olympus | 0.583405325 | 0.226401715 | 238 |
| 2 | Pu Wang 2020 | EndoScreener | 0.361855528 | 0.092982632 | 484 |
| 3 | Daisuke Yamaguchi 2023 | CAD EYE | -0.239238038 | 0.254324826 | 113 |
| 4 | Hirotaka Nakashima 2023 | CAD EYE | 0.061710331 | 0.03695299 | 207 |
| 5 | Jeremy R. Glissen Brown 2021 | EndoScreener | -0.076025744 | 0.100328931 | 113 |
| 6 | Ahmir Ahmad 2023 | GI Genius | 0.246753247 | 1.62079453 | 308 |
| 7 | Pu Wang 2019 | Wision AI | 0.394964831 | 0.120010215 | 522 |
| 8 | Ping Shen 2021 | Tianjin YuJin | 0.133101852 | 0.873950141 | 64 |
| 9 | Liwen Yao 2022 | EndoAngel | 0.131326761 | 0.042479394 | 268 |
| 10 | Peixi Liu 2020 | EndoScreener | 0.538171144 | 0.163507997 | 393 |
| 11 | Lei Xu 2021 | AI-assisted | 0.125055044 | 0.039847638 | 1177 |
| 12 | Liu WN 2019 | AI-assisted | 0.610889855 | 0.18561761 | 508 |
| 13 | Dexin Gong 2020 | EndoAngel | 0.118422858 | 0.041021309 | 355 |
|  |  |  |  |  |  |
|  |  |  |  |  |  |
| id | study id | t | mean | sd | n |
| 1 | Thomas Ka-Luen Lui 2024 | Standard Colonoscopy | 0.583405325 | 0.226401715 | 214 |
| 2 | Pu Wang 2020 | Standard Colonoscopy | 0.361855528 | 0.092982632 | 478 |
| 3 | Daisuke Yamaguchi 2023 | Standard Colonoscopy | -0.239238038 | 0.254324826 | 118 |
| 4 | Hirotaka Nakashima 2023 | Standard Colonoscopy | 0.061710331 | 0.03695299 | 208 |
| 5 | Jeremy R. Glissen Brown 2021 | Standard Colonoscopy | -0.076025744 | 0.100328931 | 110 |
| 6 | Ahmir Ahmad 2023 | Standard Colonoscopy | 0.246753247 | 1.62079453 | 306 |
| 7 | Pu Wang 2019 | Standard Colonoscopy | 0.394964831 | 0.120010215 | 536 |
| 8 | Ping Shen 2021 | Standard Colonoscopy | 0.133101852 | 0.873950141 | 54 |
| 9 | Liwen Yao 2022 | Standard Colonoscopy | 0.131326761 | 0.042479394 | 271 |
| 10 | Peixi Liu 2020 | Standard Colonoscopy | 0.538171144 | 0.163507997 | 397 |
| 11 | Lei Xu 2021 | Standard Colonoscopy | 0.125055044 | 0.039847638 | 1175 |
| 12 | Liu WN 2019 | Standard Colonoscopy | 0.610889855 | 0.18561761 | 518 |
| 13 | Dexin Gong 2020 | Standard Colonoscopy | 0.118422858 | 0.041021309 | 349 |
|  |  |  |  |  |  |
|  | Small (>5 to <10 mm) |  |  |  |  |
| id | study id | treatment | mean | sd | n |
| 1 | Thomas Ka-Luen Lui 2024 | Olympus | 0.101193749 | 0.039270191 | 238 |
| 2 | Pu Wang 2020 | EndoScreener | 0.029202255 | 0.025924513 | 484 |
| 3 | Daisuke Yamaguchi 2023 | CAD EYE | -0.255887206 | 0.583407369 | 113 |
| 4 | Hirotaka Nakashima 2023 | CAD EYE | 0.116824601 | 0.068891032 | 207 |
| 5 | Jeremy R. Glissen Brown 2021 | EndoScreener | 0.127353178 | 0.168064231 | 113 |
| 6 | Ahmir Ahmad 2023 | GI Genius | 0.013326543 | 0.012619783 | 308 |
| 7 | Pu Wang 2019 | Wision AI | 0.045197861 | 0.02275316 | 522 |
| 8 | Ping Shen 2021 | Tianjin YuJin | 0.082175926 | 0.26433867 | 64 |
| 9 | Liwen Yao 2022 | EndoAngel | 0.030222504 | 0.022798033 | 268 |
| 10 | Peixi Liu 2020 | EndoScreener | 0.024105729 | 0.025738299 | 393 |
| 11 | Lei Xu 2021 | AI-assisted | -0.020604855 | 0.00649005 | 1177 |
| 12 | Liu WN 2019 | AI-assisted | 0.047669717 | 0.023440134 | 508 |
| 13 | Dexin Gong 2020 | EndoAngel | 0.005294806 | 0.012424411 | 355 |
|  |  |  |  |  |  |
|  |  |  |  |  |  |
| id | study id | treatment | mean | sd | n |
| 1 | Thomas Ka-Luen Lui 2024 | Olympus | 0.101193749 | 0.039270191 | 214 |
| 2 | Pu Wang 2020 | EndoScreener | 0.029202255 | 0.025924513 | 478 |
| 3 | Daisuke Yamaguchi 2023 | CAD EYE | -0.255887206 | 0.583407369 | 118 |
| 4 | Hirotaka Nakashima 2023 | CAD EYE | 0.116824601 | 0.068891032 | 208 |
| 5 | Jeremy R. Glissen Brown 2021 | EndoScreener | 0.127353178 | 0.168064231 | 110 |
| 6 | Ahmir Ahmad 2023 | GI Genius | 0.013326543 | 0.012619783 | 306 |
| 7 | Pu Wang 2019 | Wision AI | 0.045197861 | 0.02275316 | 536 |
| 8 | Ping Shen 2021 | Tianjin YuJin | 0.082175926 | 0.26433867 | 54 |
| 9 | Liwen Yao 2022 | EndoAngel | 0.030222504 | 0.022798033 | 271 |
| 10 | Peixi Liu 2020 | EndoScreener | 0.024105729 | 0.025738299 | 397 |
| 11 | Lei Xu 2021 | AI-assisted | -0.020604855 | 0.00649005 | 1175 |
| 12 | Liu WN 2019 | AI-assisted | 0.047669717 | 0.023440134 | 518 |
| 13 | Dexin Gong 2020 | EndoAngel | 0.005294806 | 0.012424411 | 349 |
|  |  |  |  |  |  |
|  | Large（≥10mm） |  |  |  |  |
| id | study id | treatment | mean | sd | n |
| 1 | Thomas Ka-Luen Lui 2024 | Olympus | 0.08631116 | 0.033494714 | 238 |
| 2 | Pu Wang 2020 | EndoScreener | -0.00028528 | 0.011379625 | 484 |
| 3 | Daisuke Yamaguchi 2023 | CAD EYE | -0.08099595 | 0.085569712 | 113 |
| 4 | Hirotaka Nakashima 2023 | CAD EYE | 0.019602378 | 0.100920102 | 207 |
| 5 | Jeremy R. Glissen Brown 2021 | EndoScreener | 0.07079646 | 0.093427999 | 113 |
| 6 | Ahmir Ahmad 2023 | GI Genius | 0.082420847 | 0.067776969 | 308 |
| 7 | Pu Wang 2019 | Wision AI | 0.011994625 | 0.009736617 | 522 |
| 8 | Ping Shen 2021 | Tianjin YuJin | 0.116319444 | 0.083282025 | 64 |
| 9 | Liwen Yao 2022 | EndoAngel | 0.011317949 | 0.011193518 | 268 |
| 10 | Peixi Liu 2020 | EndoScreener | -0.004883958 | 0.009494934 | 393 |
| 11 | Lei Xu 2021 | AI-assisted | 0.00414975 | 0.006656247 | 1177 |
| 12 | Liu WN 2019 | AI-assisted | 0.012419056 | 0.00992834 | 508 |
| 13 | Dexin Gong 2020 | EndoAngel | 0.022389927 | 0.012487177 | 355 |
|  |  |  |  |  |  |
|  |  |  |  |  |  |
| id | study id | treatment | mean | sd | n |
| 1 | Thomas Ka-Luen Lui 2024 | Olympus | 0.08631116 | 0.033494714 | 214 |
| 2 | Pu Wang 2020 | EndoScreener | -0.00028528 | 0.011379625 | 478 |
| 3 | Daisuke Yamaguchi 2023 | CAD EYE | -0.08099595 | 0.085569712 | 118 |
| 4 | Hirotaka Nakashima 2023 | CAD EYE | 0.019602378 | 0.100920102 | 208 |
| 5 | Jeremy R. Glissen Brown 2021 | EndoScreener | 0.07079646 | 0.093427999 | 110 |
| 6 | Ahmir Ahmad 2023 | GI Genius | 0.082420847 | 0.067776969 | 306 |
| 7 | Pu Wang 2019 | Wision AI | 0.011994625 | 0.009736617 | 536 |
| 8 | Ping Shen 2021 | Tianjin YuJin | 0.116319444 | 0.083282025 | 54 |
| 9 | Liwen Yao 2022 | EndoAngel | 0.011317949 | 0.011193518 | 271 |
| 10 | Peixi Liu 2020 | EndoScreener | -0.004883958 | 0.009494934 | 397 |
| 11 | Lei Xu 2021 | AI-assisted | 0.00414975 | 0.006656247 | 1175 |
| 12 | Liu WN 2019 | AI-assisted | 0.012419056 | 0.00992834 | 518 |
| 13 | Dexin Gong 2020 | EndoAngel | 0.022389927 | 0.012487177 | 349 |
