# Supplementary material for: Comparative Efficacy of Different AI Systems for Polyp Detection by Size During Colonoscopy: Systematic Review and Network Meta-Analysis
Source: J Med Internet Res. 2026 Sep 9;28:e95841. doi: 10.2196/95841 (PMC13601876; doi:10.2196/95841)
Supplement: Supplementary file 8 [file jmir_v28i1e95841_app8.docx]

**Figure S1 Sensitivity‑analysis forest plot of standardized mean difference (SMD) for mean count of diminutive polyps (≤5 mm). Comparison between AI‑assisted colonoscopy and standard colonoscopy under Hartung‑Knapp‑Sidik‑Jonkman (HKSJ) random‑effects model. Positive SMD values indicate higher polyp‑detection counts in the AI‑assisted group; negative SMD values favour standard colonoscopy. Black horizontal bars represent 95% confidence intervals for individual studies; thick red horizontal line shows the 95% prediction interval. Black diamond represents pooled SMD. Heterogeneity metrics are shown below the plot.**

**Figure S2** **Sensitivity‑analysis forest plot of standardized mean difference (SMD) for mean count of small polyps (6‑9 mm). Comparison between AI‑assisted colonoscopy and standard colonoscopy under Hartung‑Knapp‑Sidik‑Jonkman (HKSJ) random‑effects model. Positive SMD values indicate higher polyp‑detection counts in the AI‑assisted group; negative SMD values favour standard colonoscopy. Black horizontal bars represent 95% confidence intervals for individual studies; thick red horizontal line shows the 95% prediction interval. Black diamond represents pooled SMD. Heterogeneity metrics are shown below the plot.**

**Figure S3 Sensitivity‑analysis forest plot of standardized mean difference (SMD) for mean count of large polyps (≥10 mm). Comparison between AI‑assisted colonoscopy and standard colonoscopy under Hartung‑Knapp‑Sidik‑Jonkman (HKSJ) random‑effects model. Positive SMD values indicate higher polyp‑detection counts in the AI‑assisted group; negative SMD values favour standard colonoscopy. Black horizontal bars represent 95% confidence intervals for individual studies; thick red horizontal line shows the 95% prediction interval. Black diamond represents pooled SMD. Heterogeneity metrics are shown below the plot.**

**Figure S4 League table of indirect comparisons for diminutive polyps (≤5 mm). Values represent standardized mean differences (95% confidence intervals) for row intervention minus column intervention. All comparisons are indirect via standard colonoscopy; no head-to-head trials exist between AI systems. Non-significant comparisons are indicated by confidence intervals crossing zero.**

**Figure S5 League table of indirect comparisons for large polyps (≥10 mm). Values represent standardized mean differences (95% confidence intervals) for row intervention minus column intervention.**

.

**Figure S6 League table of indirect comparisons for small polyps (6–9 mm). Values represent standardized mean differences (95% confidence intervals) for row intervention minus column intervention.**
